# Supplementary material for: KeyGenes, a Tool to Probe Tissue Differentiation Using a Human Fetal Transcriptional Atlas
Source: Stem Cell Reports. 2015 May 28;4(6):1112–24. doi: 10.1016/j.stemcr.2015.05.002 (PMC4472038; doi:10.1016/j.stemcr.2015.05.002)
Supplement: Document S1. Supplemental Experimental Procedures and Figures S1–S5 [file mmc1.pdf]

Stem Cell Reports, Volume *4*

Supplemental Information

KeyGenes, a Tool to Probe Tissue Differentiation Using a Human Fetal Transcriptional Atlas

Matthias S. Roost, Liesbeth van Iperen, Yavuz Ariyurek, Henk P. Buermans, Wibowo Arindrarto, Harsha D. Devalla, Robert Passier, Christine L. Mummery, Françoise Carlotti, Eelco J.P. de Koning, Erik W. van Zwet, Jelle J. Goeman, and Susana M. Chuva de Sousa Lopes

SUPPLEMENTAL FIGURES

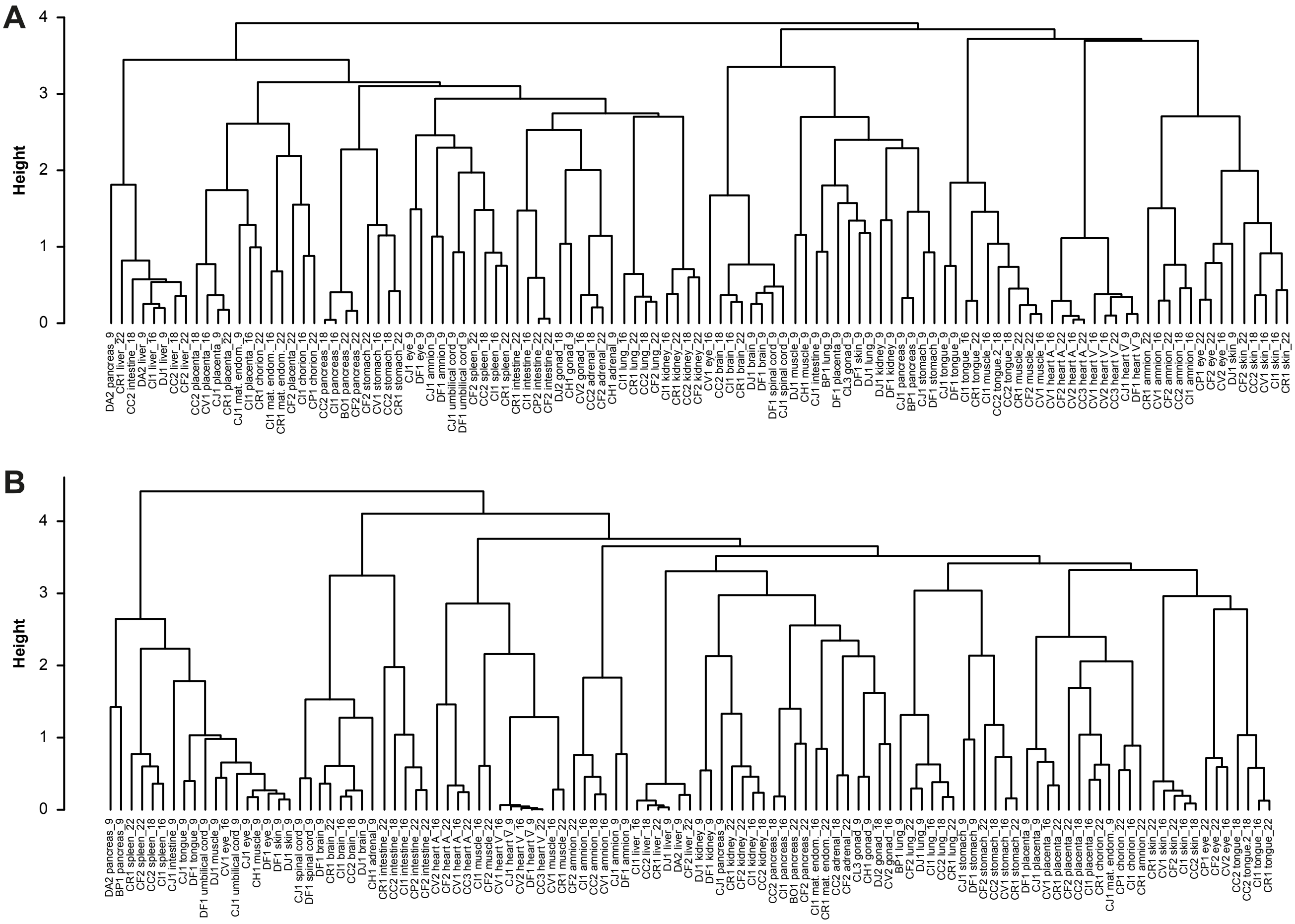

Figure S1. Hierarchical clustering of the fetal samples

(A) Dendrogram showing the hierarchical clustering of all fetal and maternal endometrium samples, based on the Pearson correlation of the expression levels of the 90 fetal classifier genes.

(B) Dendrogram showing the hierarchical clustering of all fetal and maternal endometrium samples, based on the Pearson correlation of the expression levels of the 500 most variable genes of the entire fetal data set (Top 500 fetal).

Abbreviations: mat. endom., maternal endometrium.

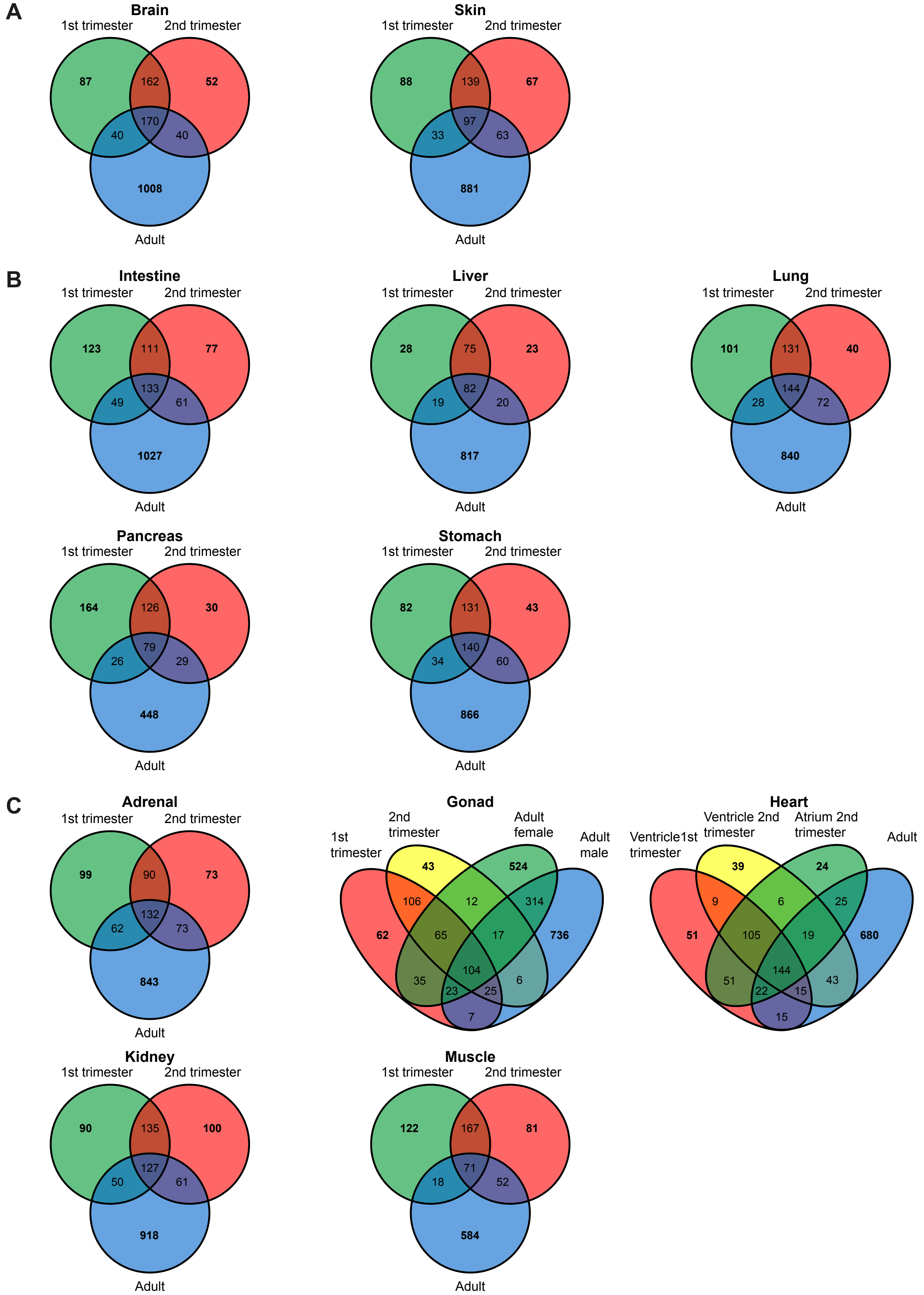

Figure S2. Stage-specific gene expression signatures per organ

Venn diagrams of commonly and uniquely highly expressed genes of the first trimester, second trimester and adult samples per organ derived mainly from (A) ectoderm, (B) endoderm, and (C) mesoderm. The genes shown here have 10x higher gene expression levels in counts per million (CPM) than the average expression of the corresponding dataset. The stage-specific genes per organ and the intersections genes are given in Table S4.

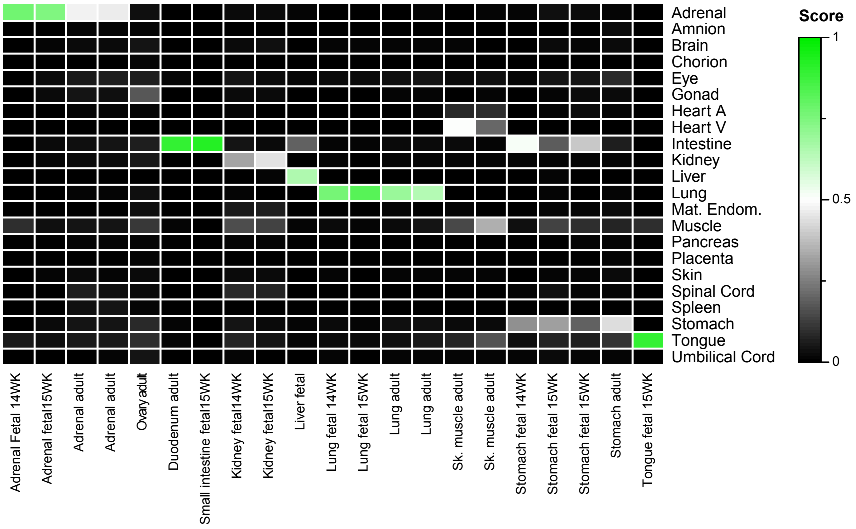

Figure S3. Prediction of the microarray human adult and fetal dataset from Nazor et al (2012)
Identity scores of 21 human adult and fetal organ/tissue samples from the microarray dataset generated by Nazor et al. (Nazor et al., 2012). The rows represent the 22 organs/tissues from the fetal training set and the columns depict the samples in the test set. The identity scores range from zero (black) to one (green). The values of all identity scores are given in Table S3.

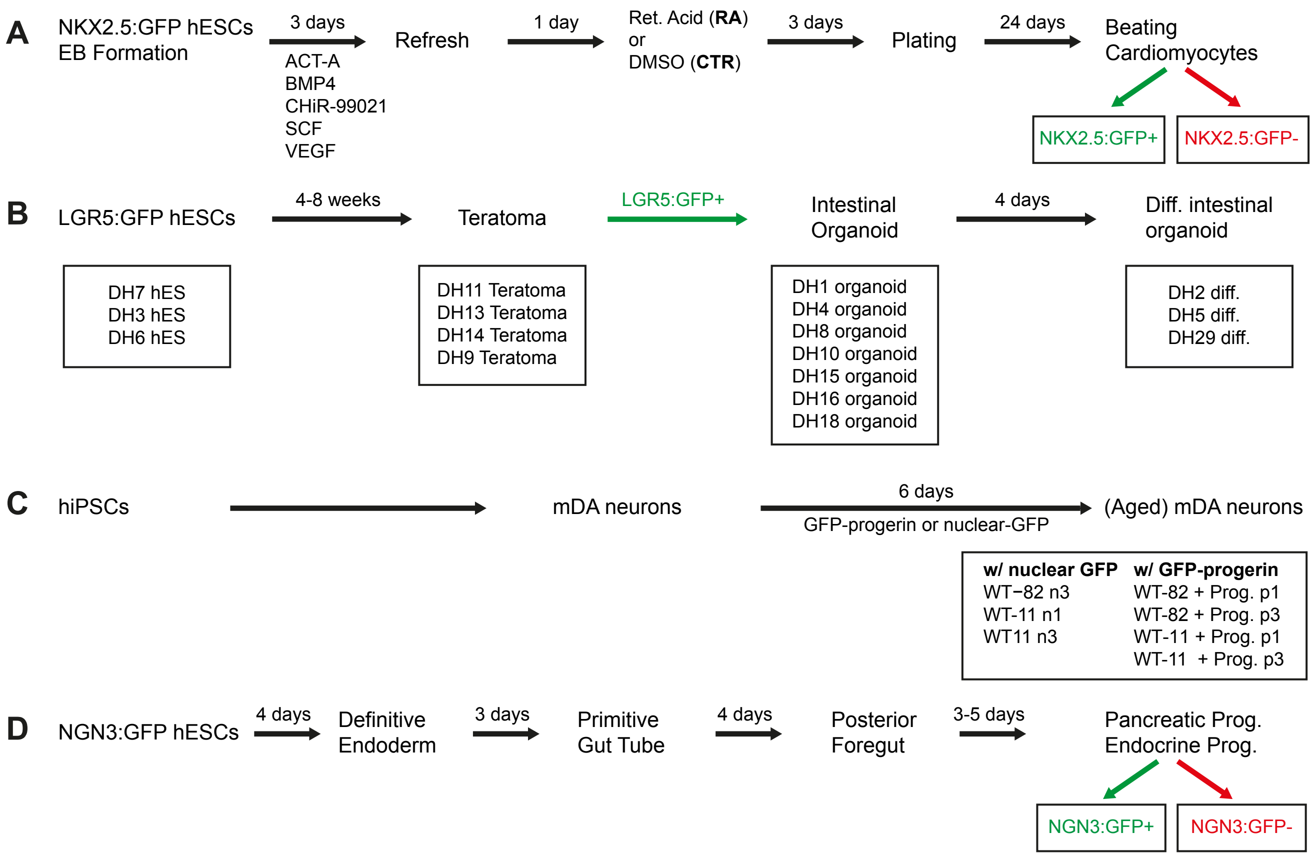

Figure S4. Schematics of the differentiation protocols used to generate hPSC-derived cardiomyocytes, intestinal organoids, dopaminergic neurons and pancreatic progenitor cells

(A) Generation of human cardiomyocytes from NKX2.5:GFP human embryonic stem cells (hESCs) (Devalla et al., 2015).

(B) Generation of intestinal organoids from LGR5:GFP human embryonic stem cells (hESCs) (Forster et al., 2014).

(C) Generation of (aged) dopaminergic neurons (mDA neurons) from human induced pluripotent stem cells (hiPSCs) (Miller et al., 2013).

(D) Generation of pancreatic progenitors from NGN3:GFP human embryonic stem cells (hESCs) (Liu et al., 2014).

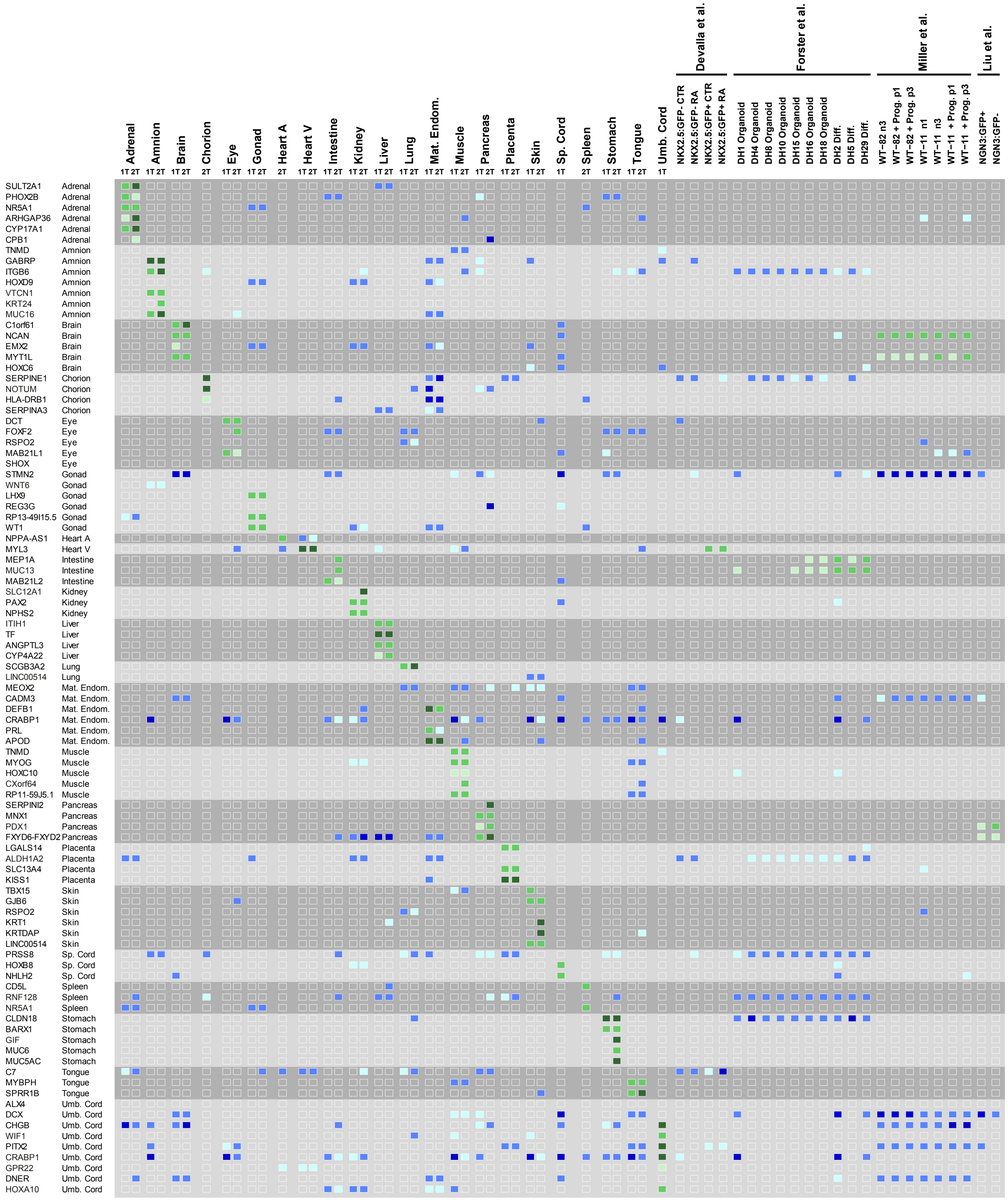

Figure S5. Expression levels of the 90 classifier genes in the hPSC-derived cardiomyocytes, intestinal organoids, dopaminergic neurons and pancreatic progenitor cells

Heatmap of the expression levels of the fetal transcriptional barcode (90 classifier genes, Figure 2) and the organ they represent during first and second trimester compared to the differentiation experiments from Figures 4 and 5 (Devalla et al., 2015; Forster et al., 2014; Liu et al., 2014; Miller et al., 2013). The expression levels (in counts per million, CPM) of the classifier genes in the corresponding tissue are depicted in green (50-100 CPM, light green; 100-1000 CPM, green; >1000 CPM, dark green), whereas the expression levels in other than the classifying tissues are displayed in blue (50-100 CPM, light blue; 100-1000 CPM, blue; >1000 CPM, dark blue).

Table S1. Characteristics of the human fetal and maternal endometrium samples

Table S2. Human fetal barcode - 90 classifier genes and the corresponding GO enrichment

Table S3. Identity scores of all test sets and the different lists of the 500 most variably expressed genes

Table S4. Specific gene expression signatures per time point per organ

SUPPLEMENTAL EXPERIMENTAL PROCEDURES

Tissue culture and immunofluorescence

Cardiomyocytes from NKX2.5:GFP hESCs were generated as described (Devalla et al., 2015) (Figure S4A). After 30 days, NKX2.5:GFP-positive cells were FACS sorted using a BD FACSAria III Cell sorter (BD Biosciences, USA) and used for library preparation. Immunofluorescence for COUP-FTII and GFP followed by imaging with confocal microscopy were described elsewhere (Devalla et al., 2015).

NGS library preparation, sequencing and data processing

The organs and tissues were homogenized using a pestle or a syringe needle followed by the QIAshredder homogenizer (Qiagen, Germany). Total RNA was isolated with the RNeasy Kit (Qiagen) including on-column DNase digestion. RNA quality was assessed with the Agilent RNA 6000 Pico or Nano Kit on an Agilent 2100 Bioanalyzer (Agilent Technologies, USA). RNA concentration was determined with the Qubit RNA BR Assay Kit on a Qubit 2.0 Fluorometer (Invitrogen, USA).

The DeepSAGE libraries (fetal organs and tissues) were generated as previously described (Mastrokolias et al., 2012). Each library (8 pM) was loaded on a v3 flowcell and sequenced on an Illumina HiSeq2000 sequencer (Illumina, USA). Sequencing data was processed with Illumina Pipeline Software version CASAVA 1.8.2, and the FASTQ files were analyzed with a set of custom Perl scripts followed by aligning the sequences against the UCSC hg19 reference genome using Bowtie short read aligner 1.0.0. Finally, the tags were annotated against Ensembl gene identifiers using BIOMART.

The strand-specific RNA-Seq libraries of the NKX2.5:GFP-positive and -negative cells were generated essentially as previously described (Parkhomchuk et al., 2009). Following amplification of the strand-specific libraries, they were paired-end sequenced (2x 75 bp) on a

single NextSeq500 Mid output lane (Illumina, USA). Base quality trimming was performed on the raw sequencing files using SICKLE 1.33. Afterwards, the sequences were aligned to a custom hg19 human genome using GSNAP release 2014-12-06. We ran GSNAP by setting the following flags: --batch (set to 4), --novelsplicing (set to 1), --npaths (set to 1), and --quiet-if-excessive. The last two flags were required so that reads map if and only if it can be mapped to a unique location in the genome. The resulting SAM file was then compressed into a BAM file, name-sorted, and indexed using SAMTOOLS 0.1.18. Finally, gene read count data were generated using HTSeq-count 0.6.1p1 using the Ensembl reference downloaded via the UCSC website (http://genome.ucsc.edu), using the non-stranded and intersection-nonempty option flags (--stranded no and --m intersection-nonempty respectively).

External Data

For the Illumina Bodymap dataset, we performed gene read counting using HTSeq-count version 0.6.1p1 using the Ensembl human genes annotation set downloaded from the UCSC website (http://genome.ucsc.edu) in December 2014. We used the non-stranded and intersection-nonempty option flags (--stranded no and --m intersection-nonempty respectively) as additional arguments to the program. As the Illumina BodyMap 2.0 data were comprised of single-end and paired-end libraries, we separated them first and performed read counting on the separated library.

SUPPLEMENTAL REFERENCES

Devalla, H.D., Schwach, V., Ford, J.W., Milnes, J.T., El-Haou, S., Jackson, C., Gkatzis, K., Elliott, D.A., Chuva de Sousa Lopes, S.M., Mummery, C.L., *et al.* (2015). Atrial-like cardiomyocytes from human pluripotent stem cells are a robust preclinical model for assessing atrial-selective pharmacology. EMBO Mol Med *7*, 394-410.

Forster, R., Chiba, K., Schaeffer, L., Regalado, S.G., Lai, C.S., Gao, Q., Kiani, S., Farin, H.F., Clevers, H., Cost, G.J., *et al.* (2014). Human intestinal tissue with adult stem cell properties derived from pluripotent stem cells. Stem cell reports *2,* 838-852.

Liu, H., Yang, H., Zhu, D., Sui, X., Li, J., Liang, Z., Xu, L., Chen, Z., Yao, A., Zhang, L., *et al.* (2014). Systematically labeling developmental stage-specific genes for the study of pancreatic beta-cell differentiation from human embryonic stem cells. Cell research *24,* 1181-1200.

Mastrokolias, A., den Dunnen, J.T., van Ommen, G.B., t Hoen, P.A., and van Roon-Mom, W.M. (2012). Increased sensitivity of next generation sequencing-based expression profiling after globin reduction in human blood RNA. BMC genomics *13*, 28.

Miller, J.D., Ganat, Y.M., Kishinevsky, S., Bowman, R.L., Liu, B., Tu, E.Y., Mandal, P.K., Vera, E., Shim, J.W., Kriks, S., *et al.* (2013). Human iPSC-based modeling of late-onset disease via progerin-induced aging. Cell stem cell *13,* 691-705.

Nazor, K.L., Altun, G., Lynch, C., Tran, H., Harness, J.V., Slavin, I., Garitaonandia, I., Muller, F.J., Wang, Y.C., Boscolo, F.S., *et al.* (2012). Recurrent variations in DNA methylation in human pluripotent stem cells and their differentiated derivatives. Cell stem cell *10,* 620-634.

Parkhomchuk, D., Borodina, T., Amstislavskiy, V., Banaru, M., Hallen, L., Krobitsch, S., Lehrach, H., and Soldatov, A. (2009). Transcriptome analysis by strand-specific sequencing of complementary DNA. Nucleic acids research *37,* e123.
