# Supplementary material for: KeyGenes, a Tool to Probe Tissue Differentiation Using a Human Fetal Transcriptional Atlas
Source: Stem Cell Reports. 2015 May 28;4(6):1112–24. doi: 10.1016/j.stemcr.2015.05.002 (PMC4472038; doi:10.1016/j.stemcr.2015.05.002)
Supplement: Document S2. Article plus Supplemental Information [file mmc6.pdf]

Stem Cell Reports
Resource
OPEN ACCESS

KeyGenes, a Tool to Probe Tissue Differentiation Using a Human Fetal Transcriptional Atlas

Matthias S. Roost,[1] Liesbeth van Iperen,[1] Yavuz Ariyurek,[2] Henk P. Buermans,[2] Wibowo Arindrarto,[3] Harsha D. Devalla,[1] Robert Passier,[1] Christine L. Mummery,[1] Françoise Carlotti,[5] Eelco J.P. de Koning,[5,6] Erik W. van Zwet,[4] Jelle J. Goeman,[7] and Susana M. Chuva de Sousa Lopes[1,8,*]
[1]Department of Anatomy and Embryology
[2]Leiden Genome Technology Center
[3]Sequence Analysis Support Core
[4]Department of Medical Statistics and Bioinformatics
Leiden University Medical Center, Einthovenweg 20, 2333 ZC Leiden, the Netherlands
[5]Department of Nephrology, Leiden University Medical Center, Albinusdreef 2, 2333 ZA Leiden, the Netherlands
[6]Hubrecht Institute, Uppsalalaan 8, 3584 CT Utrecht, the Netherlands
[7]Department for Health Evidence, Radboud University Medical Center, Geert Grooteplein 21, 6525 EZ Nijmegen, the Netherlands
[8]Department for Reproductive Medicine, Ghent University Hospital, De Pintelaan 185, 9000 Ghent, Belgium
*Correspondence: lopes@lumc.nl
http://dx.doi.org/10.1016/j.stemcr.2015.05.002
This is an open access article under the CC BY license (http://creativecommons.org/licenses/by/4.0/).

SUMMARY

Differentiated derivatives of human pluripotent stem cells in culture are generally phenotypically immature compared to their adult counterparts. Their identity is often difficult to determine with certainty because little is known about their human fetal equivalents in vivo. Cellular identity and signaling pathways directing differentiation are usually determined by extrapolating information from either human adult tissue or model organisms, assuming conservation with humans. To resolve this, we generated a collection of human fetal transcriptional profiles at different developmental stages. Moreover, we developed an algorithm, KeyGenes, which uses this dataset to quantify the extent to which next-generation sequencing or microarray data resemble specific cell or tissue types in the human fetus. Using KeyGenes combined with the human fetal atlas, we identified multiple cell and tissue samples unambiguously on a limited set of features. We thus provide a flexible and expandable platform to monitor and evaluate the efficiency of differentiation in vitro.

INTRODUCTION

Detailed information on temporal and spatial patterns of gene expression during human development is essential to understand how cells establish and maintain their transcriptional identity and how they differentiate from common progenitors to form the different organs in the human body. Moreover, knowledge of the gene expression landscape in a physiological context is of paramount importance to identify aberrant patterns of transcription leading to pathological states (Ju et al., 2013; Lal et al., 1999). To date, there is little information on the transcriptional profiles of organs and tissues during human development, even though optimal use of differentiated derivatives of human pluripotent stem cells (hPSCs) in regenerative medicine and disease modeling would benefit from detailed understanding of what drives and maintains the differentiated state. This knowledge is not only helpful in developing efficient differentiation protocols for hPSCs and unequivocally identifying the resultant phenotypes (Gifford et al., 2013; Xie et al., 2013), but also in understanding why individual (genomic) variations among hPSCs may result in different outcomes (Bock et al., 2011) and whether the wide assumption that some disease states are associated with upregulation of fetal genes is actually based on fact (Fung et al., 2012; Hoshijima and Chien, 2002; Lin et al., 2014).

Recently, there has been great interest in computational approaches that help to quantify differences in physiological and pathological states in human adult organs and tissues (Hwang et al., 2011) and similarities between adult human organs/tissues and differentiated cells derived from either hPSCs or by direct lineage conversion, also known as transdifferentiation (Cahan et al., 2014; Morris et al., 2014). The drawback of these computational approaches so far is that they have been based on adult human organs/tissues, which limits their relevance in assigning developmental states to differentiated derivatives of hPSCs or transdifferentiated somatic cells, since they are often immature and resemble fetal cells more than those of mature adult tissues (Hrvatin et al., 2014; Patterson et al., 2012). Moreover, most current computational tools use and compare to microarray datasets only. Therefore, there is a growing need for a computational platform that can integrate next-generation sequencing (NGS) and microarray datasets and facilitate their interrogation.

Here we present an algorithm, KeyGenes, that we have used on NGS data extracted from tissues of 21 different human fetal organs, both embryonic and extraembryonic (plus the maternal endometrium), from the first and

second trimesters of development to determine a panel of classifier genes that would be sufficient to confer identity to each fetal organ analyzed with high confidence. We showed that the developmental classifier genes selected were largely sufficient to predict the identity of their adult organ counterparts, even when using different types of platforms (NGS and microarray). Most importantly, as proof of concept, we challenged KeyGenes to identify a series of tissues using either recently published or our own NGS datasets. These included the following: (1) hPSCs differentiated to derivatives of the three germ lineages, namely, endoderm (pancreas), ectoderm (brain), and mesoderm (heart); (2) tissue organoids (intestine); and (3) human fetal and adult organs/tissues. In all cases, KeyGenes accurately predicted tissue origin and, furthermore, we could use KeyGenes to assign a developmentally equivalent stage. KeyGenes is an easy-to-use, flexible, and expandable tool that can be applied to identify stem cell derivatives, when common marker profiles have been insufficiently informative, and provide benchmarking for protocols designed to promote maturation of stem cell derivatives in culture. KeyGenes is available at http://www.keygenes.nl.

RESULTS

KeyGenes Defines a Transcriptional Barcode to Predict Human Fetal Organ Identity

We wanted to identify a (sex-independent) transcriptional barcode that would characterize each organ primordium during human fetal development, as these are still relatively homogeneous and mainly formed by a limited number of poorly characterized lineage progenitor cells.

To do this, we first generated NGS data from 111 human organ/tissue samples from 17 individuals (n = 9 males and n = 8 females) representing 21 different fetal organs, 17 embryonic and 4 extraembryonic (plus the maternal endometrium), at 8.2–9.6, 16–18, and 21–22 weeks of gestation (W9, W16–18, and W22) (Table S1). Next, we developed an algorithm (KeyGenes) that uses a 10-fold crossvalidation on the basis of a least absolute shrinkage and selection operator (LASSO) regression available in the R package "glmnet" (Friedman et al., 2010; Figure 1A). KeyGenes uses two datasets (a training set and a test set) and one table with the 500 most variably expressed genes. Then, KeyGenes determines a set of classifier genes that is sufficient to identify the samples of the training set, and, finally, using the classifier genes, it predicts the identity of the samples of the test set. To validate KeyGenes, we divided our fetal dataset into a training set (n = 76) and a test set (n = 39) (Figures 1A and 1B), so that at least two samples of each organ, preferentially of both first and second trimesters, were represented. Based on the classifier genes (Table S2) and the identity of the samples in the training set, KeyGenes then predicted the identity of the samples in the test set (Figure 1C). KeyGenes was capable of predicting 38 of 39 samples of the test set correctly with a mean identity score of 0.92, of which 33 samples (87%) had an identity score >0.8 (Table S3). KeyGenes thus provided a highly accurate prediction of fetal tissue identity for the test fetal samples.

The Transcriptional Barcode for Human Fetal Organs during Development

A common characteristic of the 90 fetal classifier genes identified (Table S2) is that these are expressed at high levels throughout development in the organ(s)/tissue(s) they characterize (in general >100 counts per million [CPM], but some >1,000 CPM), and they are either not expressed in most other tissues or highly expressed in several other tissues simultaneously, helping to define the barcode (Figure 2A). Interestingly, large differences in expression levels of some classifier genes were even sufficient to distinguish between first and second trimester organs. For example, *CYP17A1* in the adrenal is expressed >100 CPM during the first trimester, but >1,000 CPM in the second trimester (Figure 2A). Five classifier genes (*TNMD*, *RSPO2*, *LINC00514*, *NR5A1*, and *CRABP1*) identified two different fetal organs/tissues (Table S2). The selected classifiers for each organ/tissue varied from one gene (*NPPA-AS1* for heart atrium and *MYL3* for heart ventricle) to nine genes for the umbilical cord (Figure 2B).

In terms of gene ontology (GO), it is noteworthy that the fetal classifier genes identified were sufficiently related to be significantly enriched (false discovery rate [FDR] < 0.05) for categories associated with biological processes ("patterning," "morphogenesis," and "development"), cellular compartment ("extracellular"), and molecular function ("transcription" and "DNA binding") (Figure 2C; Table S2). Furthermore, a substantial proportion of the fetal classifier genes are transcription factors (n = 24) (Figure 2D), many being tissue specific and considered master regulator genes in mice (Table S2). However, it was striking that about half of the total fetal classifier genes were in fact not directly related to transcription, but were instead associated with extracellular matrix (ECM), cell adhesion, and surface tension or were components of the cytoskeleton and cellular transport machinery (Figure 2D; Table S2). This underscored the importance of cellular shape, structure, and the niche in determining tissue identity and function. Interestingly, a few classifier genes are long non-coding RNAs (lncRNAs) or anti-sense RNAs (asRNAs) (n = 4), including the unique classifier gene for heart atrium (*NPPA-AS1*). Our data are in agreement with the growing evidence placing lncRNAs as an emerging class

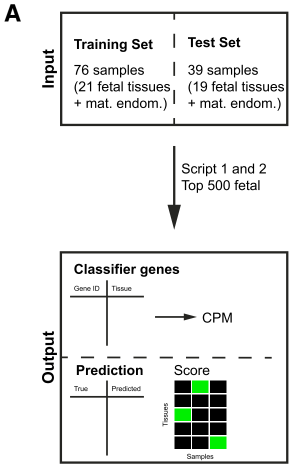

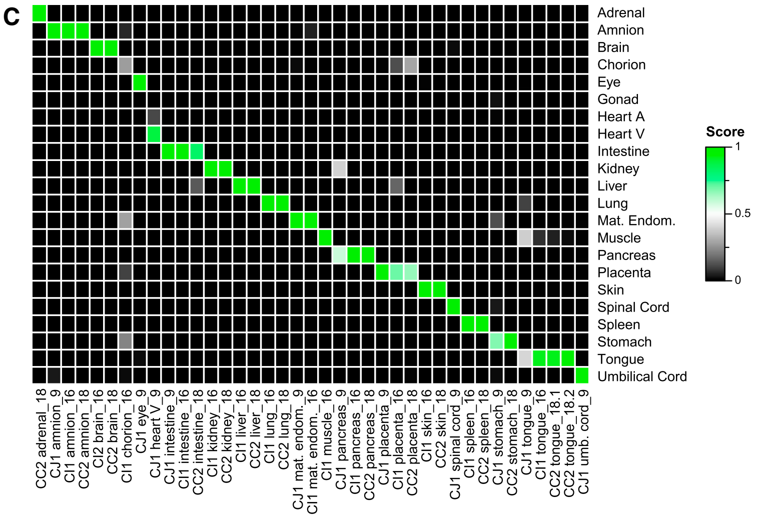

B

		Extraembryonic				Ectoderm				Endoderm					Mesoderm								Other
		Amnion	Chorion	Placenta	Umb. Cord	Brain	Eye	Skin	Sp. Cord	Intestine	Liver	Lung	Pancreas	Stomach	Adrenal	Gonad	Heart A	Heart V	Kidney	Sk. Muscle	Spleen	Tongue	Mat. Endom.
Training Set (n=76)	1T	1	0	1	2	2	1	2	2	1	2	2	2	1	1	2	0	1	2	2	0	1	1
	2T	3	2	3	0	1	4	3	0	3	2	2	2	3	1	2	4	3	2	3	2	1	1
Test Set (n=39)	1T	1	0	1	1	0	1	0	1	1	0	0	1	1	0	0	0	1	0	0	0	1	1
	2T	2	1	2	0	2	0	2	0	2	2	2	2	1	1	0	0	0	2	1	2	3	1

Figure 1. Validation of KeyGenes Using a Human Fetal Transcriptome Dataset

(A) The fetal dataset was divided into a training set and a test set. KeyGenes used the 500 most variably expressed genes across the entire fetal dataset (top 500 fetal) to determine a panel of classifier genes. KeyGenes then used the classifier genes to predict the identity (and provide the identity score) of the samples in the test set.

(B) Characteristics of the human fetal samples in the training set and test set are shown.

(C) Identity scores for the samples in the fetal test set. The rows represent the 22 organs/tissues from the fetal training set and the columns depict the samples in the test set. The identity scores range from zero (black) to one (green). The values of all identity scores are given in Table S3. 1T, first trimester; 2T, second trimester; heart A, heart atrium; heart V, heart ventricle; mat. endom., maternal endometrium; sk. muscle, skeletal muscle; sp. cord, spinal cord; umb. cord, umbilical cord.

of important cellular regulators in development (Washietl et al., 2014).

Hierarchical clustering (Pearson correlation) of the expression values per organ and trimester (1T + 2T), as well as per organ independent of the trimester (All), resulted in a higher mean correlation coefficient using the 90 classifier genes than the mean correlation coefficient that was derived using the 500 most variably expressed genes of the entire fetal dataset (Top 500 fetal) (Figure 2E). Accordingly, the clustering of the fetal samples using the 90 classifier genes was sufficient to group them in a germ layer-, tissue-, and age-specific manner (Figures S1A and S1B). Taken together, our results suggested that the classifier genes, identified as the minimal set of genes or barcode necessary to predict the identity of 21 different fetal human organs and the maternal endometrium, represent physiologically relevant genes, many of which are associated with human pathological conditions affecting the organ/tissue they characterize (Table S2).

Using the Fetal Data, KeyGenes Predicts Human Adult Tissue Identity from NGS Data

We next investigated whether the fetal transcriptional barcode could be used to predict the identity of adult human tissues. To this end, we applied KeyGenes to two different NGS datasets of human adult organs as test sets, using our complete human fetal dataset (111 samples representing 21 fetal tissues and the maternal endometrium) as the training set (Figure 3A) and the top 500 most variable fetal genes determined previously (Table S3).

KeyGenes was first applied to an NGS dataset consisting of 61 samples representing adult counterparts of 17 organs present in our fetal training set (Fagerberg et al., 2014). Surprisingly, 56 samples (92% of all adult samples) were predicted correctly with a high mean identity score of 0.90 (Figure 3B; Table S3). This suggested that the degree of inter-individual variation between biopsies of the same organ is remarkably low. More importantly, adult tissue biopsies of 17 different organs retained significant similarities to

fetal organs with respect to the basic transcriptional wiring, independently of cellular heterogeneity in the adult organ (Figure 3B). Even though five samples (two of skin, one of term placenta, two of cervix) were not predicted correctly, their correctly predicted biological replicates showed high identity scores (one skin, three term placenta, one cervix), and the second best score of those misclassified samples pointed to the correct organ/tissue (Figure 3B; Table S3). Further illustrating the predictive power of KeyGenes, three of the four heart samples were classified as "heart ventricles" (mean identity score = 0.93) and were probably ventricular biopsies, whereas one heart sample was assigned a score of 0.39 for "heart atrium" and 0.4 for "heart ventricle," suggesting that this sample may have contained both ventricular and atrial heart tissue (Figure 3B; Table S3).

Additionally, we used a second resource of NGS data from biopsies of ten human adult organs (Human Body Map 2.0, Illumina), which were represented in our fetal training set. From this, KeyGenes predicted nine of ten samples (90%) correctly with a mean identity score of 0.90 (Figure 3C; Table S3). The one misclassified sample was the adrenal gland, which was classified as "spleen" (0.72). However, the previous adult dataset contained three biological replicates of adrenal samples, all correctly classified with a mean identity score of 0.99 (Figure 3B; Table S3), suggesting that the adrenal gland sample present in the Human Body Map 2.0 by Illumina may in fact not be adrenal.

We have shown that KeyGenes together with the provided fetal dataset is a platform capable of predicting the identity of adult counterparts of fetal tissues with high confidence. Moreover, our results suggest that the minimal transcriptional organ barcode identified during human fetal development is in fact maintained into adulthood, regardless of the increasing cellular complexity of each organ. In addition, we have presented the differentially expressed genes among the first trimester, second trimester, and adult for each organ analyzed, showing the transcriptional trajectory of maturation for the tissue/organs analyzed (Figure S2; Table S4).

Using the Fetal Data, KeyGenes Predicts Human Adult Tissue Identity from Microarray Data

Although the use of NGS is of increasing importance, DNA microarray technology is still widely used as it provides fast and relatively inexpensive gene expression data. Because of the fundamentally different nature of data from NGS and the relative (fluorescence) intensity from microarray, we adapted our algorithm to process microarray data (Figure 3A). To do this, KeyGenes used the fetal dataset as training set and the 500 most variably expressed fetal genes determined before (Top 500 fetal) (Table S3), and it contained a scaling step using a broad panel of housekeeping genes (Eisenberg and Levanon, 2013).

We applied KeyGenes to 53 samples, mostly human adult organs, tissues, and cell subpopulations, available online from the Gene Expression Barcode 3.0 (Affymetrix; McCall et al., 2014), which were represented in our fetal training set. KeyGenes was able to predict 45 of 53 samples (85%) correctly with a high mean identity score of 0.86 (Figure 3D; Table S3), suggesting a successful adaptation of the algorithm.

Two of the mismatched organs were the adult ovary and testis, but both tissues were predicted with high confidence as "gonad" before using both NGS datasets (Figures 3B and 3C), suggesting that the probes of important classifier genes for "gonad" may not be well represented on the microarray. Consequently, the sample "sperm" also was not well predicted. It would be interesting to see whether NGS data from this very specialized population of cells would still be identified as "gonad." Another misclassified organ was the pancreas, which was predicted as "intestine," but was predicted correctly using NGS data (Figure 3B), suggesting that the probes for pancreatic classifier genes were probably not well represented on the microarray. The sample "heart ventricle" had a very similar mixed "atrium and ventricle" prediction to one of the NGS heart biopsies (Figure 3B). Finally, the two tongue muscles included in the microarray datasets were identified as "skin" instead of "tongue" or "muscle"; and the only spinal cord sample was identified as "brain" instead of "spinal cord." As we were unable to find available NGS from both adult tongue and spinal cord, we could not conclude whether the mismatch was due to a lack of representation of classifier genes on the microarray or a genuine mismatch between the expression profile between the fetal and adult organ.

It is noteworthy that microarray data from specific subpopulations of cells or tissues of the adult organs also were assigned correctly to the tissue of origin. The 15 samples from different anatomical regions related to "brain" were all classified correctly, and sub-regions of the intestine (four sub-regions), kidney (four sub-regions), and stomach (four sub-regions) also were all assigned to the correct main organ (Figure 3D). Moreover, the sample adult lung and fetal lung were predicted as "lung" with an identity score of 0.91 and 0.98, respectively, whereas the bronchus and the bronchial epithelial cells had a "lung" prediction but a lower identity score (0.77 and 0.65, respectively), suggesting a better match with the full tissue than with sub-populations (Figure 3D; Table S3).

Furthermore, we analyzed predictions in a second microarray dataset from a different platform (Illumina; Nazor et al., 2012). KeyGenes was capable of predicting 18 of 21 relevant samples (86%) correctly, with a relatively high mean identity score of 0.6 (Figure S3; Table S3). The mean identity score of the correctly predicted fetal tissues

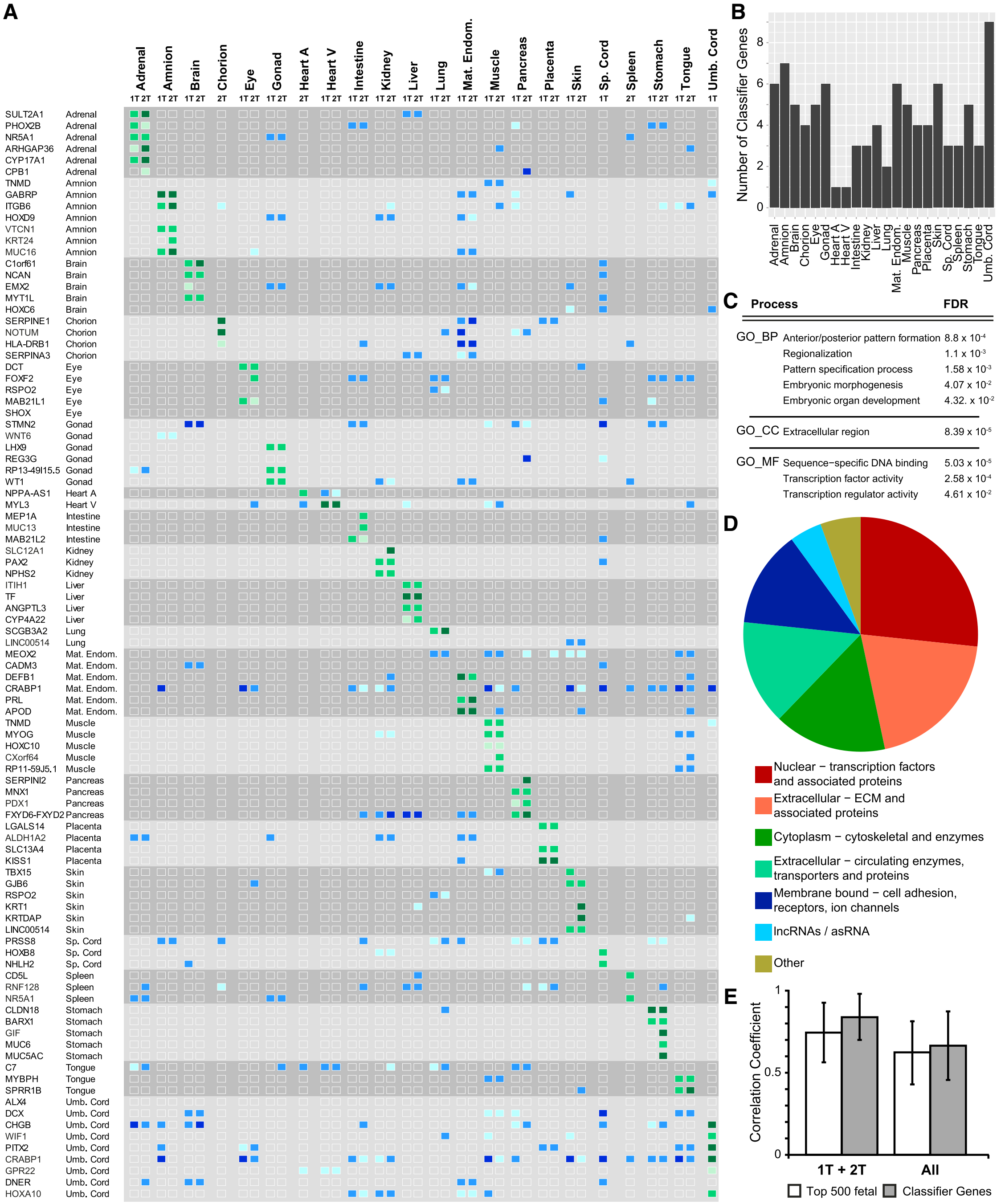

Figure 2. KeyGenes Provides a Transcriptional Barcode for First and Second Trimesters of Human Fetal Development

(A) Heatmap of the expression levels of the fetal transcriptional barcode (90 classifier genes) and the organs they represent during the first (1T) and second (2T) trimesters. The expression levels (in CPM) of the classifier genes in the corresponding organ/tissue are depicted in green (50–100 CPM, light green; 100–1,000 CPM, green; >1,000 CPM, dark green), whereas the expression levels in other than the classifying tissues are displayed in blue (50–100 CPM, light blue; 100–1,000 CPM, blue; >1,000 CPM, dark blue). All organs/tissues per trimester show a unique expression pattern of the classifier genes.

(B) Numbers of fetal classifier genes per organ/tissue used by KeyGenes to predict identity of the samples in the fetal test set are shown.

(C) Enrichment of GO terms for biological processes (BP), cellular compartment (CC), and molecular function (MF) with an FDR < 0.05 of the 90 fetal classifier genes. All GO terms are provided in Table S2.

(D) Categorization of the 90 fetal classifier genes by CC and MF is given.

(legend continued on next page)

(n = 12) was 0.67, whereas it was 0.52 for the adult tissues (n = 7) (Figure S3; Table S3).

In summary, our algorithm is not only robust enough for NGS or microarray data, but it is also capable of assigning correct organ predictions to datasets from specific parts of adult human organs, suggesting that each organ's transcriptional basic wiring or barcode identified by KeyGenes from the fetal set remains stable until adulthood, at least with respect to the 18 main adult organs analyzed. The intersection of the fetal classifier genes obtained for each of the four predictions ("fetal from fetal" and "adult from fetal") resulted in 71 common genes, suggesting that these may be the most relevant genes (represented as well on the microarray platform) to characterize the basic wiring of both human fetal and adult organs (Figure 3E; Table S2).

Monitoring Differentiation Efficiency from hPSCs with KeyGenes

One challenging issue in stem cell research is still how to reliably determine the identity and extent of differentiation of stem cells toward a specific cell or tissue type. To determine whether KeyGenes was suitable for this purpose, we used it on multiple NGS datasets that represented hPSCs differentiated to four different tissue/cell types. We adapted both the fetal training set and calculated the top 500 most variably expressed genes, excluding the extraembryonic fetal tissues and the maternal endometrium (Figure 4A; Table S3).

First, we generated atrial-like and ventricular-like cardiomyocytes (Figure S4A) from an NKX2.5:GFP human embryonic stem cell (hESC) reporter line (Elliott et al., 2011) and assessed their signature with KeyGenes. Cardiac differentiation of hESCs resulted predominantly in GFP-positive cells with ventricular-like identity, while exogenous treatment with retinoic acid (RA) during differentiation resulted in GFP-positive cells with an atrial-like identity, as shown by the upregulation of atrial markers (*COUP-TFII*) and downregulation of ventricular markers (*MYL3*) much like the atria and ventricles of the fetal heart (Figure 4B; Devalla et al., 2015). Furthermore, atrial identity of RA-treated cardiomyocytes also was demonstrated at the protein level by co-expression of the atrial marker COUP-TFII and GFP (Figure 4C). Using KeyGenes, the control NKX2.5:GFP-positive hESC cardiomyocytes showed a high identity score with heart ventricle (Figure 4D), while, in the RA-treated group, NKX2.5:GFP-positive hESC cardiomyocytes increased their identity score for "heart atrium" (from 0.06 to 0.15), although these cells were still predicted to be "heart ventricle" but with a lower identity score (reduced from 0.75 to 0.40) (Table S3). Interestingly, in the fetal barcode matrix, RA-treated NKX2.5:GFP-positive hESC cardiomyocytes showed similar *MYL3* expression levels as atrial cardiomyocytes, but lacked expression of *NPPA-AS1* (Figures 4B and S5).

Next, we used a dataset from LGR5:GFP reporter hESCs that were first induced to form teratomas in mice (Forster et al., 2014; Figure S4B); from the teratomas, "adult intestinal stem cell"-like cells were sorted via fluorescence-activated cell sorting (FACS) on the basis of GFP fluorescence intensity, and then induced to form organoids resembling intestinal tissue before being differentiated further. We analyzed the NGS data from different time points during the differentiation. Using our fetal dataset as training set, nine of ten organoids (organoids and differentiated organoids) were classified as "intestine" with a mean identity score 0.85 (Figure 4E; Table S3). We also included the NGS data of undifferentiated hESCs in the analysis and, interestingly, the highest identity score was for "brain," suggesting that this ectodermal tissue during fetal development remains relatively immature. Furthermore, KeyGenes detected a large difference between different organoids, but not a large difference in maturation levels between the organoids and their differentiated counterparts, underscoring the ability of KeyGenes to monitor differentiation and indicate developmental stage equivalents.

Assigning Developmental Stages to Differentiated hPSC Derivatives with KeyGenes

To examine the broader applicability of KeyGenes in assigning developmental stages to differentiated hPSC derivatives, we analyzed NGS data from dopaminergic neurons derived by the differentiation of human induced pluripotent stem cells (hiPSCs) from old (82 years) and young (11 years) donors (Miller et al., 2013). These dopaminergic neurons were then either transfected with GFP-progerin, a protein involved in premature aging, or nuclear GFP as control (Figure S4C). These aged neurons are regarded as being possibly useful as a platform to investigate late-onset diseases such as Parkinson's disease. All seven samples analyzed were classified as "brain" with a mean score of 0.47 (Figure 5A; Table S3). KeyGenes confirmed that the aging of the neurons by progerin had a clear impact on differentiation, always assigning a higher identity score for "brain" to progerin-transfected neurons (mean score of

(E) Mean Pearson correlation coefficient (±SD) between samples of the same organ based on the expression levels of the 500 most variably expressed genes (top 500 fetal) or the 90 fetal classifier genes. The mean correlation coefficient was higher using the 90 fetal classifier genes, both when the trimester was taken into account (1T + 2T) and when samples were considered regardless of the trimester (all). Abbreviations are as given in Figure 1.

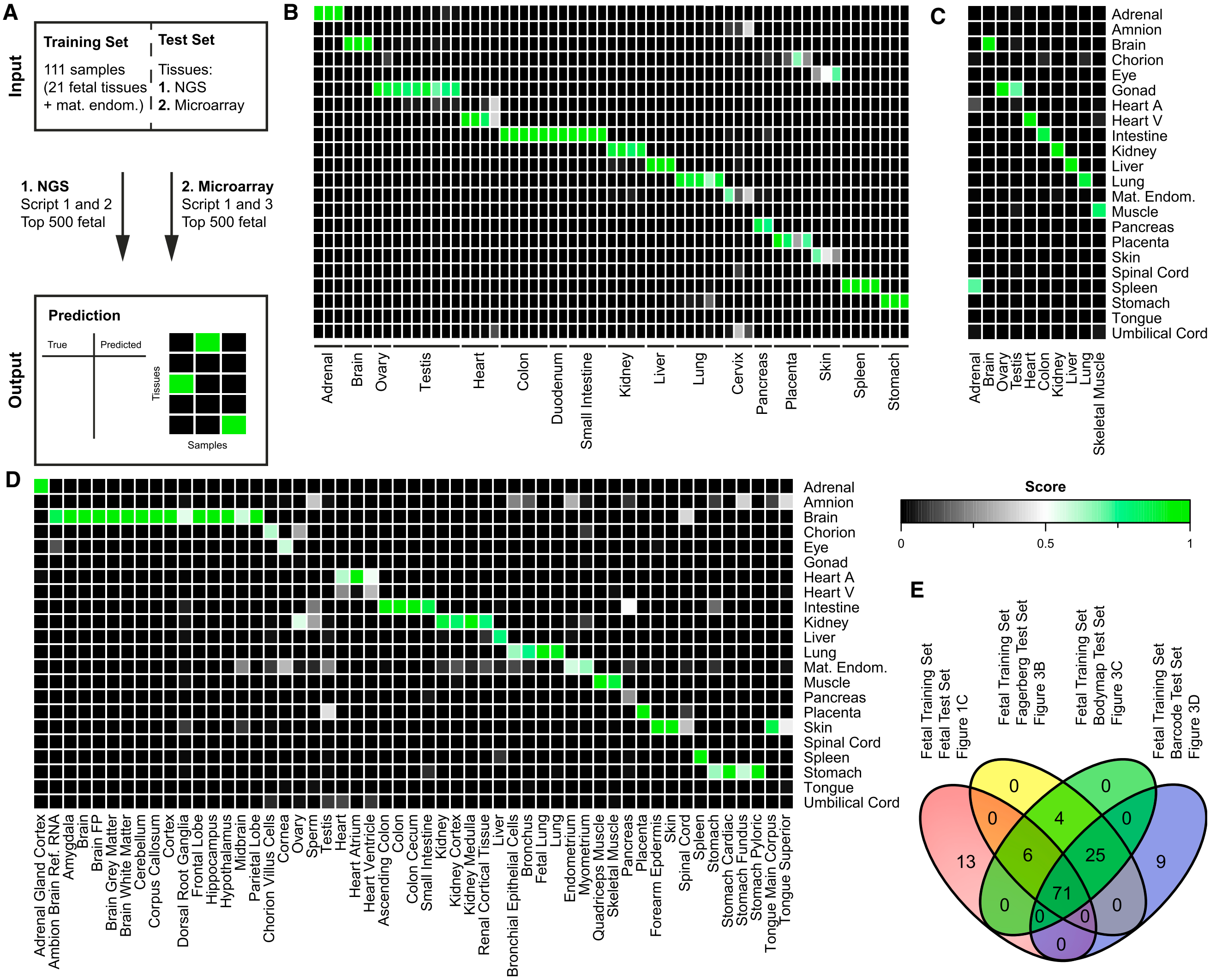

Figure 3. KeyGenes Classifies Human Adult Organs and Tissues Based on the Fetal Transcriptome

(A) KeyGenes was applied to human adult NGS and microarray datasets (test sets) using the fetal dataset as training set. (1) For NGS-derived count data, KeyGenes searches for the best classifier genes out of the 500 most variable genes of the fetal dataset (Top 500 fetal). (2) For microarray-derived data, KeyGenes searches for the best classifier genes out of the Top 500 fetal and applies a scaling step to a broad panel of housekeeper genes (Eisenberg and Levanon, 2013).

(B–D) Identity scores of 17 human adult organ/tissue samples from an NGS dataset (Fagerberg et al., 2014) (B), of ten human adult organ/tissue samples from the NGS dataset from the Illumina Body Map 2.0 (C), and of 53 human adult organ/tissue samples from the microarray dataset in Gene Expression Barcode 3.0 (McCall et al., 2014) (D). The rows represent the 22 organs/tissues from the fetal training set and the columns depict the samples in the test set. The identity scores range from zero (black) to one (green). The values of all identity scores are given in Table S3.

(E) Venn diagram shows the intersection of the classifier fetal genes used by KeyGenes to identify/predict the different test sets (fetal NGS test set in Figure 1C; adult NGS test set in (B) [Fagerberg et al., 2014]; adult NGS test set from the Illumina Body Map 2.0 in (C); and adult microarray test set from the Gene Expression Barcode 3.0 in (D) [McCall et al., 2014]). The 71 common fetal classifier genes are depicted in Table S2. Abbreviations are as given in Figure 1.

0.57) than to controls (mean score of 0.35) (Figure 5A; Table S3). When the fetal training set was divided into first and second trimesters (Figure 5B) with the corresponding top 500 most variably expressed genes, we observed that the samples had higher identity scores with first trimester "brain" tissue than with second trimester "brain" tissue. Moreover, using adult samples in the training set resulted in lower identity scores (Figure 5C) than when either the

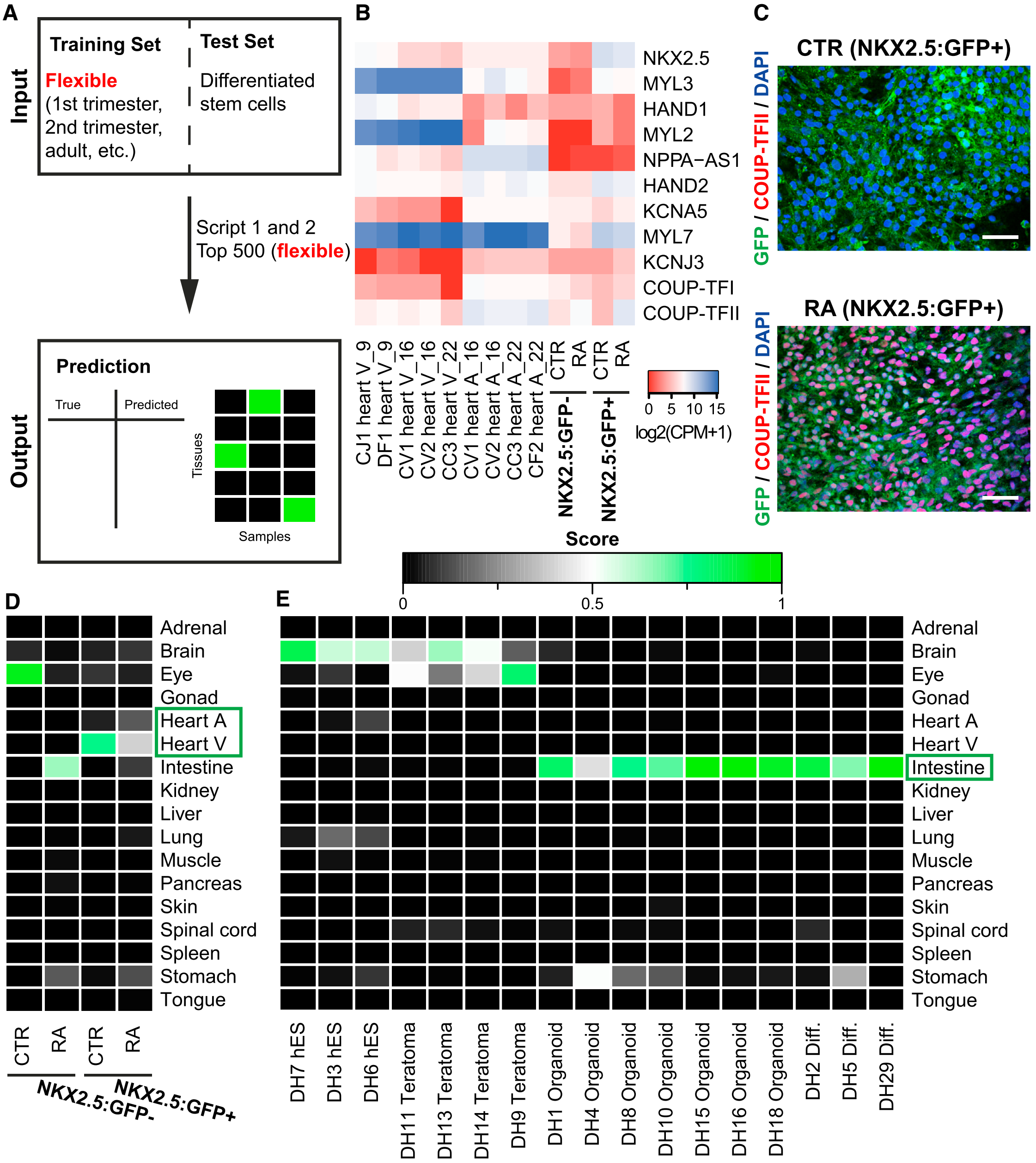

Figure 4. KeyGenes to Monitor hPSC Differentiation

(A) KeyGenes was applied to NGS datasets from differentiated derivatives of hPSCs (test set) using flexible training sets. The selection of the classifier genes is based on the 500 most variable genes across a given dataset and can be modified.

(B) Heatmap visualizes the binary logarithm of the expression levels (CPM) of selected atrial and ventricular marker genes in the human fetal heart atrium (A) and ventricle (V) samples and in the control (CTR) and RA-differentiated NKX2.5:GFP-positive and -negative cells.

(C) Immunofluorescence shows COUP-TFII in the CTR and RA-differentiated NKX2.5:GFP-positive subpopulation. Scale bar, 40 μm.

(D and E) Identity scores of the CTR and RA-treated NKX2.5:GFP-positive and -negative cells (D) and of hESC samples, teratomas, intestinal organoids derived from hESCs, and organoids that have been differentiated further toward intestine (diff.) (Forster et al., 2014) (E). The fetal dataset was used as training set excluding the extraembryonic tissues and the maternal endometrium samples. The selection of the classifier genes is based on the 500 most variable genes of the embryonic training set without extraembryonic and maternal endometrium samples (top 500 fetal w/o). The rows represent the 17 organs/tissues from the fetal training set and the columns depict the samples in the test set. The identity scores range from zero (black) to one (green). The values of all identity scores are given in Table S3. Abbreviations are as given in Figure 1.

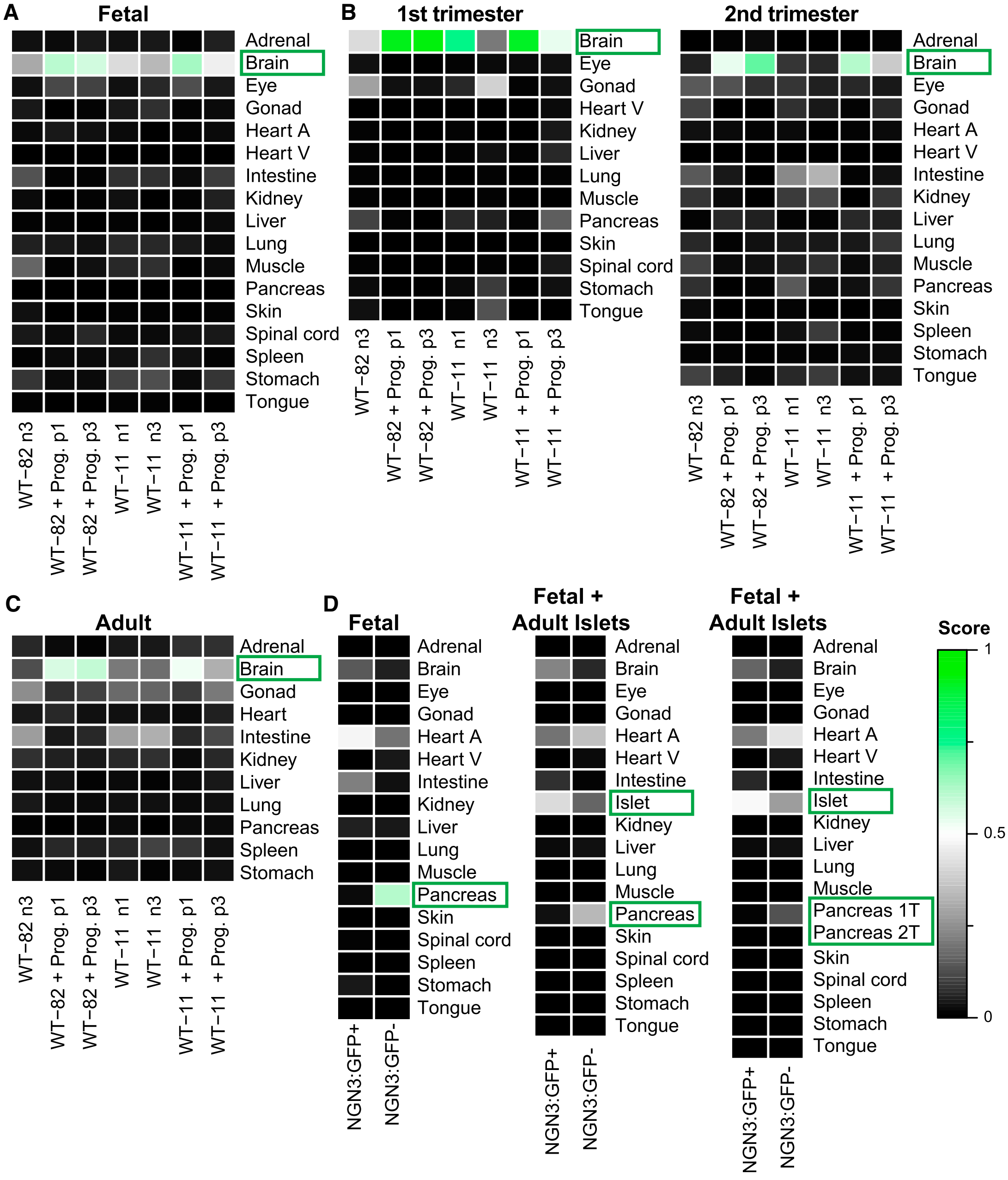

Figure 5. KeyGenes Can Be Modified to Assign Developmental Stages

(A–C) Identity scores of dopaminergic neurons derived from two different hiPSC lines, WT-82 and WT-11 (Miller et al., 2013). The dopaminergic neurons were further differentiated by transfection with progerin-GFP (prog.) or nuclear GFP as a control. As training set, KeyGenes used either the whole fetal dataset excluding the extraembryonic tissues and the maternal endometrium samples (A), only the first trimester or second trimester samples excluding the extraembryonic tissues and the maternal endometrium samples (B), or the adult NGS datasets (Fagerberg et al., 2014; Illumina Bodymap) (C) with the corresponding 500 most variable genes (top 500 fetal w/o, top 500 fetal w/o 1T, top 500 fetal w/o 2T, and top 500 adult).

(D) Identity scores of NGN3:GFP-positive and NGN3:GFP-negative cells derived from hESCs (Liu et al., 2014). The fetal training set excluding the extraembryonic tissues and the maternal endometrium samples was expanded by an NGS dataset consisting of five human adult islet of Langerhans samples (Cnop et al., 2014). The 500 most variable genes of the embryonic training set without extraembryonic and maternal endometrium samples (top 500 fetal w/o) were used. The rows represent the organs/tissues from the different training sets and the columns depict the samples in the test set. The identity scores range from zero (black) to one (green). The values of all identity scores are given in Table S3. Abbreviations are as given in Figure 1.

fetal training set or the first and second trimester samples were used separately. This suggested that, even though the progerin-samples were predicted as being "brain," the identity score compared with adult brain was even lower. Using each training set, we noted that there were evident differences in identity scores between cells that had undergone different numbers of passage in culture (Figures 5A–5C). This showed how KeyGenes can be used to monitor differentiation conditions as well as to compare replicates to improve protocol outcomes.

Finally, we applied KeyGenes to NGS data from pancreatic endocrine progenitor cells differentiated from NGN3:eGFP hESCs (Liu et al., 2014; Figure S4D). *NGN3* is an essential transcription factor during pancreas development, specifying the fate of its endocrine cells (Rukstalis and Habener, 2009). A multi-step differentiation protocol was used to differentiate NGN3:GFP hESCs toward pancreatic β cell lineage; cells were then sorted via FACS for GFP and analyzed at the endocrine progenitor stage (Figure S4D). The NGN3:GFP-negative differentiated population was classified as "pancreas" using the fetal training set, confirming the presence of pancreatic exocrine (progenitor) cells (Figure 5D; Table S3). Interestingly, the NGN3:GFP-positive differentiated population, presumably containing the endocrine progenitors, was misclassified as "heart atrium" (Figure 5D; Table S3). However, large proportions of the pancreas are in fact exocrine cells and the endocrine cells contribute only 1%–2% to the pancreatic cell mass (Chu et al., 2001; Rahier et al., 1981). This also explains why the classifier genes for the (fetal) pancreas are mainly genes involved in exocrine function of the pancreas (Figure 2A; Table S2).

Therefore, to have meaningful predictions with respect to the endocrine lineage (NGN3:GFP-positive differentiated population), the fetal training data used by KeyGenes were enriched by an available NGS dataset consisting of five human adult pancreatic islets (Cnop et al., 2014). Using the 500 most variably expressed genes across our fetal training set, excluding the extraembryonic fetal tissues and the maternal endometrium (Table S3), the misclassification of the NGN3:GFP-positive cell population disappeared and was predicted as "islet" with a score of 0.41 (Figure 5D; Table S3). Additionally, by separating first- and second-trimester pancreas (1T and 2T) in the training set, the identity score for the NGN3:GFP-positive cell population increased for "islet" to 0.49 (Figure 5D; Table S3). This highlights the importance of being able to implement the appropriate training set, depending on the purpose of each differentiation experiment, to be able to assign meaningful identity scores and determine the equivalent developmental stage (either by using a different training set for each stage or by separating the developmental stages of the tissue/cells of interest in the same training set) to improve the outcomes of the differentiation protocols.

DISCUSSION

We have shown here through detailed genomic analysis that human organs and tissues retain a transcriptional signature from W9 until adulthood even though each organ is composed of multiple progenitor cell types that mature over time. It was remarkable that the transcriptional expression profile of a set of less than 100 genes was sufficient to identify 21 different human fetal organs/tissues (plus maternal endometrium) and 18 adult human organs. These classifier genes that we identified not only included genes involved in transcription regulation but also genes that define cellular shape and metabolism. Moreover, some of the classifier genes were lncRNAs and asRNAs, highlighting the regulatory importance of this class of genes (Washietl et al., 2014). This was notably illustrated by *NPPA-AS1*, which is thought to regulate *NPPA* expression, a gene that encodes atrial natriuretic factor and is involved in heart development and chamber specification (Annilo et al., 2009; Houweling et al., 2005). This identification also underscores one of the advantages of KeyGenes, developed to compare data to an NGS (fetal) training set, in contrast to existing algorithms (Cahan et al., 2014; Hwang et al., 2011; Morris et al., 2014) that compare data to networks deduced from microarray datasets, which contain a fixed set of probes with low representation of non-coding RNAs such as lncRNAs and asRNAs. In our case, three of the four lncRNAs identified as fetal classifier genes (RP13-49I15.5, NPPA-AS1, and LINC00514) were not present in the microarray adult dataset, but were present in the NGS adult dataset and identified there as well as classifier genes for predictions.

The transcriptional human fetal atlas dataset presented here, even though limited in number of samples and organs analyzed, is an unique resource that will provide a deeper understanding of the signaling cascades and molecular dynamics during human development that lead into the maturation of progenitor cells within each human organ. The human fetal NGS dataset was paramount to the development and validation of our prediction algorithm KeyGenes, and has proven sufficient as training set to identify both human adult organs (using NGS and microarray data) as well as several differentiated derivatives of hPSCs. All hPSC derivatives expressed genes identified as fetal classifier genes (and many helper classifier genes) of the specific tissue to which they were claimed to have differentiated (Figure S5). Comparing differentiated derivatives of hPSCs to human adult gene expression data is important, but can be misleading, as it compares immature cells with far later

stages of development when entirely different physiological parameters have affected cell behavior. Our fetal dataset, used alone or in combination with, for example, adult data as a training set, provided an efficient way to assess progression of hPSC differentiation.

One important feature of the algorithm is that the ever-growing number of NGS datasets available online, for example, of different organs, (FACS) cell types, or simply more biological replicates, can be incorporated easily in the training set as we have demonstrated. The predictive possibilities therefore can be extended not only to organs but, as exemplified here, also to tissues (intestinal organoids) and specific cell types (cardiomyocytes, dopaminergic neurons, and pancreatic endocrine cells). These will become even more refined as more datasets become available and incorporated.

We have described and validated a valuable resource to the community that will help to determine genes important for identifying cell types and their stages of development, so that protocols for enhancing lineage differentiation efficiency or cell maturation will have accurate benchmarks to monitor the process. In addition, by incorporating similar data from human fetuses with congenital defects or derivatives of diseased patient-derived hiPSCs, it may be easier to identify the underlying molecular mechanism for the pathology.

EXPERIMENTAL PROCEDURES

Fetal Tissue Procurement

This study was approved by the Medical Ethical Committee of the Leiden Medical University Center (P08.087). Informed consent was obtained and the study was conducted in accordance with the Declaration of Helsinki by the World Medical Association. Human fetal organ and tissue samples (n = 111), from 17 individuals representing 21 organs, and maternal endometrium, between gestational W8.2 and W22 (Table S1), were obtained from elective abortion material (vacuum aspiration) without medical indication. After washing with 0.9% NaCl (Fresenius Kabi), the organs and tissues were snap-frozen in buffer RLT (QIAGEN) and stored at −80°C until further use. The organs and tissues were sex genotyped using primers for AMELOGENIN (Nakahori et al., 1991), as described previously (Heeren et al., 2015).

External Data

Gene expression data were obtained either from the Gene Expression Omnibus (GEO) database (GEO: GSE54879 [Liu et al., 2014], GSE52431 [Miller et al., 2013], GSE56930 [Forster et al., 2014], and GSE53949 [Cnop et al., 2014]) or from the EMBL-EBI database (EMBL-EBI: E-MTAB-513 [Illumina Body Map] and E-MTAB-1733 [Fagerberg et al., 2014]). The microarray data were downloaded from the Gene Expression Barcode 3.0 (http://barcode.luhs.org; McCall et al., 2014) or from the GEO database (GEO: GSE30652; Nazor et al., 2012).

Bioinformatics

KeyGenes Algorithm

The algorithm uses a 10-fold crossvalidation on the basis of a LASSO regression available in the R package “glmnet” (Friedman et al., 2010). To use KeyGenes, to access the R scripts used here, and to access extra information on the human fetal data or the training sets used here, please go to http://www.keygenes.nl. We used three R scripts as follows: (1) script 1 was used to determine the 500 most variably expressed genes across an NGS dataset (top 500), which was saved as a .txt file and then used in either script 2 or 3; (2) script 2 uses an NGS training set to predict an NGS test set, and it uses the file with the 500 most variably expressed genes generated with script 1; and (3) script 3 uses an NGS training set to predict a microarray test set, and it uses the file with the 500 most variably expressed genes generated with script 1. Script 3 is identical to script 2 but it includes a scaling step to a broad panel of 3,787 housekeeper genes (Eisenberg and Levanon, 2013). For this, the algorithm looks for the housekeeper genes that are present in the training set and the test set and chooses those that are expressed in at least one tissue.

Gene Expression Levels

As detection limit, a cutoff value of four reads was used. To evaluate the CPM expression levels, our fetal data and the tested differentiation experiments were analyzed with the R package edgeR 3.2.4, using the weighted trimmed mean of M values (TMM) method to normalize (Robinson et al., 2010; Robinson and Smyth, 2008).

Hierarchical Clustering

Hierarchical clustering using complete linkage was based on the Pearson correlation of the gene expression levels (CPM) calculated using the base R package stats.

GO Analyses

The enrichment of GO terms for the 90 classifier genes was tested with DAVID (Huang da et al., 2009). An FDR cutoff of 0.05 was used.

Venn Diagrams for Gene Expression

The datasets containing the first trimester fetal samples (1T), the second trimester fetal samples (2T), and the adult samples (Fagerberg et al., 2014; Illumina Bodymap) were compared per organ. The genes with gene expression levels higher than 10× the mean expression of the corresponding dataset (1T, 2T, adult) were assigned either unique or common appearance across the datasets per organ and visualized in a Venn diagram (Bardou et al., 2014).

Visualization

The data were primarily visualized using the R package’s gplots and ggplot2 (Warnes et al., 2014; Wickham, 2009).

ACCESSION NUMBERS

The accession numbers for the data from the fetal organs and the cardiac differentiation reported in this paper are GEO: GSE66302 and GSE67866, respectively.

SUPPLEMENTAL INFORMATION

Supplemental Information includes Supplemental Experimental Procedures, five figures, and four tables and can be found

with this article online at http://dx.doi.org/10.1016/j.stemcr.2015.05.002.

AUTHOR CONTRIBUTIONS

M.S.R., C.L.M., R.P., F.C., E.J.P.d.K., and S.M.C.d.S.L. conceived the study. M.S.R., L.v.I., Y.A., H.P.B., W.A., J.J.G., H.D.D., E.W.v.Z., and S.M.C.d.S.L conducted experiments and/or performed bioinformatic analysis. All authors were involved in analysis of the data. All authors read and approved the final manuscript.

ACKNOWLEDGMENTS

We thank H. Locher for help with the fetal dissection; M. Gomes Fernandes for the sex genotyping; G.M. de Roo for FACS of the NKX2.5:GFP cells; S.A.J de Zeeuw for bioinformatics support; and the Centre for Contraception, Abortion and Sexuality (CASA) in Leiden and the Hague for the collection of the human fetal material. S.M.C.d.S.L. is supported by the Netherlands Organisation for Scientific Research (NWO, ASPASIA 015.007.037) and the Interuniversity Attraction Poles (IAP, P7/07). M.S.R., C.L.M., and F.C. are supported by the Bontius Stichting (PANCREAS). E.J.P.d.K. is supported by Stichting DON.

Received: April 20, 2015
Revised: May 1, 2015
Accepted: May 1, 2015
Published: May 28, 2015

REFERENCES

Annilo, T., Kepp, K., and Laan, M. (2009). Natural antisense transcript of natriuretic peptide precursor A (NPPA): structural organization and modulation of NPPA expression. BMC Mol. Biol. *10*, 81.

Bardou, P., Mariette, J., Escudié, F., Djemiel, C., and Klopp, C. (2014). jvenn: an interactive Venn diagram viewer. BMC Bioinformatics *15*, 293.

Bock, C., Kiskinis, E., Verstappen, G., Gu, H., Boulting, G., Smith, Z.D., Ziller, M., Croft, G.F., Amoroso, M.W., Oakley, D.H., et al. (2011). Reference Maps of human ES and iPS cell variation enable high-throughput characterization of pluripotent cell lines. Cell *144*, 439–452.

Cahan, P., Li, H., Morris, S.A., Lummertz da Rocha, E., Daley, G.Q., and Collins, J.J. (2014). CellNet: network biology applied to stem cell engineering. Cell *158*, 903–915.

Chu, K., Nemoz-Gaillard, E., and Tsai, M.J. (2001). BETA2 and pancreatic islet development. Recent Prog. Horm. Res. *56*, 23–46.

Cnop, M., Abdulkarim, B., Bottu, G., Cunha, D.A., Igoillo-Esteve, M., Masini, M., Turatsinze, J.V., Griebel, T., Villate, O., Santin, I., et al. (2014). RNA sequencing identifies dysregulation of the human pancreatic islet transcriptome by the saturated fatty acid palmitate. Diabetes *63*, 1978–1993.

Devalla, H.D., Schwach, V., Ford, J.W., Milnes, J.T., El-Haou, S., Jackson, C., Gkatzis, K., Elliott, D.A., Chuva de Sousa Lopes, S.M., Mummery, C.L., et al. (2015). Atrial-like cardiomyocytes from human pluripotent stem cells are a robust preclinical model for assessing atrial-selective pharmacology. EMBO Mol. Med. *7*, 394–410.

Eisenberg, E., and Levanon, E.Y. (2013). Human housekeeping genes, revisited. Trends Genet. *29*, 569–574.

Elliott, D.A., Braam, S.R., Koutsis, K., Ng, E.S., Jenny, R., Lagerqvist, E.L., Biben, C., Hatzistavrou, T., Hirst, C.E., Yu, Q.C., et al. (2011). NKX2-5(eGFP/w) hESCs for isolation of human cardiac progenitors and cardiomyocytes. Nat. Methods *8*, 1037–1040.

Fagerberg, L., Hallström, B.M., Oksvold, P., Kampf, C., Djureinovic, D., Odeberg, J., Habuka, M., Tahmasebpoor, S., Danielsson, A., Edlund, K., et al. (2014). Analysis of the human tissue-specific expression by genome-wide integration of transcriptomics and antibody-based proteomics. Mol. Cell. Proteomics *13*, 397–406.

Forster, R., Chiba, K., Schaeffer, L., Regalado, S.G., Lai, C.S., Gao, Q., Kiani, S., Farin, H.F., Clevers, H., Cost, G.J., et al. (2014). Human intestinal tissue with adult stem cell properties derived from pluripotent stem cells. Stem Cell Reports *2*, 838–852.

Friedman, J., Hastie, T., and Tibshirani, R. (2010). Regularization Paths for Generalized Linear Models via Coordinate Descent. J. Stat. Softw. *33*, 1–22.

Fung, F.K., Chan, D.W., Liu, V.W., Leung, T.H., Cheung, A.N., and Ngan, H.Y. (2012). Increased expression of PITX2 transcription factor contributes to ovarian cancer progression. PLoS ONE *7*, e37076.

Gifford, C.A., Ziller, M.J., Gu, H., Trapnell, C., Donaghey, J., Tsankov, A., Shalek, A.K., Kelley, D.R., Shishkin, A.A., Issner, R., et al. (2013). Transcriptional and epigenetic dynamics during specification of human embryonic stem cells. Cell *153*, 1149–1163.

Heeren, A.M., van Iperen, L., Klootwijk, D.B., de Melo Bernardo, A., Roost, M.S., Gomes Fernandes, M.M., Louwe, L.A., Hilders, C.G., Helmerhorst, F.M., van der Westerlaken, L.A., and Chuva de Sousa Lopes, S.M. (2015). Development of the follicular basement membrane during human gametogenesis and early folliculogenesis. BMC Dev. Biol. *15*, 4.

Hoshijima, M., and Chien, K.R. (2002). Mixed signals in heart failure: cancer rules. J. Clin. Invest. *109*, 849–855.

Houweling, A.C., van Borren, M.M., Moorman, A.F., and Christoffels, V.M. (2005). Expression and regulation of the atrial natriuretic factor encoding gene Nppa during development and disease. Cardiovasc. Res. *67*, 583–593.

Hrvatin, S., O'Donnell, C.W., Deng, F., Millman, J.R., Pagliuca, F.W., DiIorio, P., Rezania, A., Gifford, D.K., and Melton, D.A. (2014). Differentiated human stem cells resemble fetal, not adult, β cells. Proc. Natl. Acad. Sci. USA *111*, 3038–3043.

Huang da, W., Sherman, B.T., and Lempicki, R.A. (2009). Systematic and integrative analysis of large gene lists using DAVID bioinformatics resources. Nat. Protoc. *4*, 44–57.

Hwang, P.I., Wu, H.B., Wang, C.D., Lin, B.L., Chen, C.T., Yuan, S., Wu, G., and Li, K.C. (2011). Tissue-specific gene expression templates for accurate molecular characterization of the normal physiological states of multiple human tissues with implication in development and cancer studies. BMC Genomics *12*, 439.

Ju, W., Greene, C.S., Eichinger, F., Nair, V., Hodgin, J.B., Bitzer, M., Lee, Y.S., Zhu, Q., Kehata, M., Li, M., et al. (2013). Defining cell-type specificity at the transcriptional level in human disease. Genome Res. *23*, 1862–1873.

Lal, A., Lash, A.E., Altschul, S.F., Velculescu, V., Zhang, L., McLendon, R.E., Marra, M.A., Prange, C., Morin, P.J., Polyak, K., et al.

(1999). A public database for gene expression in human cancers. Cancer Res. *59*, 5403–5407.

Lin, Q., Aihara, A., Chung, W., Li, Y., Huang, Z., Chen, X., Weng, S., Carlson, R.I., Wands, J.R., and Dong, X. (2014). LRH1 as a driving factor in pancreatic cancer growth. Cancer Lett. *345*, 85–90.

Liu, H., Yang, H., Zhu, D., Sui, X., Li, J., Liang, Z., Xu, L., Chen, Z., Yao, A., Zhang, L., et al. (2014). Systematically labeling developmental stage-specific genes for the study of pancreatic β-cell differentiation from human embryonic stem cells. Cell Res. *24*, 1181–1200.

McCall, M.N., Jaffee, H.A., Zelisko, S.J., Sinha, N., Hooiveld, G., Irizarry, R.A., and Zilliox, M.J. (2014). The Gene Expression Barcode 3.0: improved data processing and mining tools. Nucleic Acids Res. *42*, D938–D943.

Miller, J.D., Ganat, Y.M., Kishinevsky, S., Bowman, R.L., Liu, B., Tu, E.Y., Mandal, P.K., Vera, E., Shim, J.W., Kriks, S., et al. (2013). Human iPSC-based modeling of late-onset disease via progerin-induced aging. Cell Stem Cell *13*, 691–705.

Morris, S.A., Cahan, P., Li, H., Zhao, A.M., San Roman, A.K., Shivdasani, R.A., Collins, J.J., and Daley, G.Q. (2014). Dissecting engineered cell types and enhancing cell fate conversion via CellNet. Cell *158*, 889–902.

Nakahori, Y., Hamano, K., Iwaya, M., and Nakagome, Y. (1991). Sex identification by polymerase chain reaction using X-Y homologous primer. Am. J. Med. Genet. *39*, 472–473.

Nazor, K.L., Altun, G., Lynch, C., Tran, H., Harness, J.V., Slavin, I., Garitaonandia, I., Müller, F.J., Wang, Y.C., Boscolo, F.S., et al. (2012). Recurrent variations in DNA methylation in human pluripotent stem cells and their differentiated derivatives. Cell Stem Cell *10*, 620–634.

Patterson, M., Chan, D.N., Ha, I., Case, D., Cui, Y., Van Handel, B., Mikkola, H.K., and Lowry, W.E. (2012). Defining the nature of human pluripotent stem cell progeny. Cell Res. *22*, 178–193.

Rahier, J., Wallon, J., and Henquin, J.C. (1981). Cell populations in the endocrine pancreas of human neonates and infants. Diabetologia *20*, 540–546.

Robinson, M.D., and Smyth, G.K. (2008). Small-sample estimation of negative binomial dispersion, with applications to SAGE data. Biostatistics *9*, 321–332.

Robinson, M.D., McCarthy, D.J., and Smyth, G.K. (2010). edgeR: a Bioconductor package for differential expression analysis of digital gene expression data. Bioinformatics *26*, 139–140.

Rukstalis, J.M., and Habener, J.F. (2009). Neurogenin3: a master regulator of pancreatic islet differentiation and regeneration. Islets *1*, 177–184.

Warnes, G.R., Bolker, B., Bonebakker, L., Gentleman, R., Huber, W., Liaw, A., Lumley, T., Maechler, M., Magnusson, A., Moeller, S., et al. (2014). gplots: Various R programming tools for plotting data. R package version *2.14.2*. http://cran.r-project.org/web/packages/gplots/gplots.pdf.

Washietl, S., Kellis, M., and Garber, M. (2014). Evolutionary dynamics and tissue specificity of human long noncoding RNAs in six mammals. Genome Res. *24*, 616–628.

Wickham, H. (2009). ggplot2: Elegant Graphics for Data Analysis (New York: Springer).

Xie, W., Schultz, M.D., Lister, R., Hou, Z., Rajagopal, N., Ray, P., Whitaker, J.W., Tian, S., Hawkins, R.D., Leung, D., et al. (2013). Epigenomic analysis of multilineage differentiation of human embryonic stem cells. Cell *153*, 1134–1148.

Stem Cell Reports, Volume *4*

Supplemental Information

KeyGenes, a Tool to Probe Tissue Differentiation Using a Human Fetal Transcriptional Atlas

Matthias S. Roost, Liesbeth van Iperen, Yavuz Ariyurek, Henk P. Buermans, Wibowo Arindrarto, Harsha D. Devalla, Robert Passier, Christine L. Mummery, Françoise Carlotti, Eelco J.P. de Koning, Erik W. van Zwet, Jelle J. Goeman, and Susana M. Chuva de Sousa Lopes

SUPPLEMENTAL FIGURES

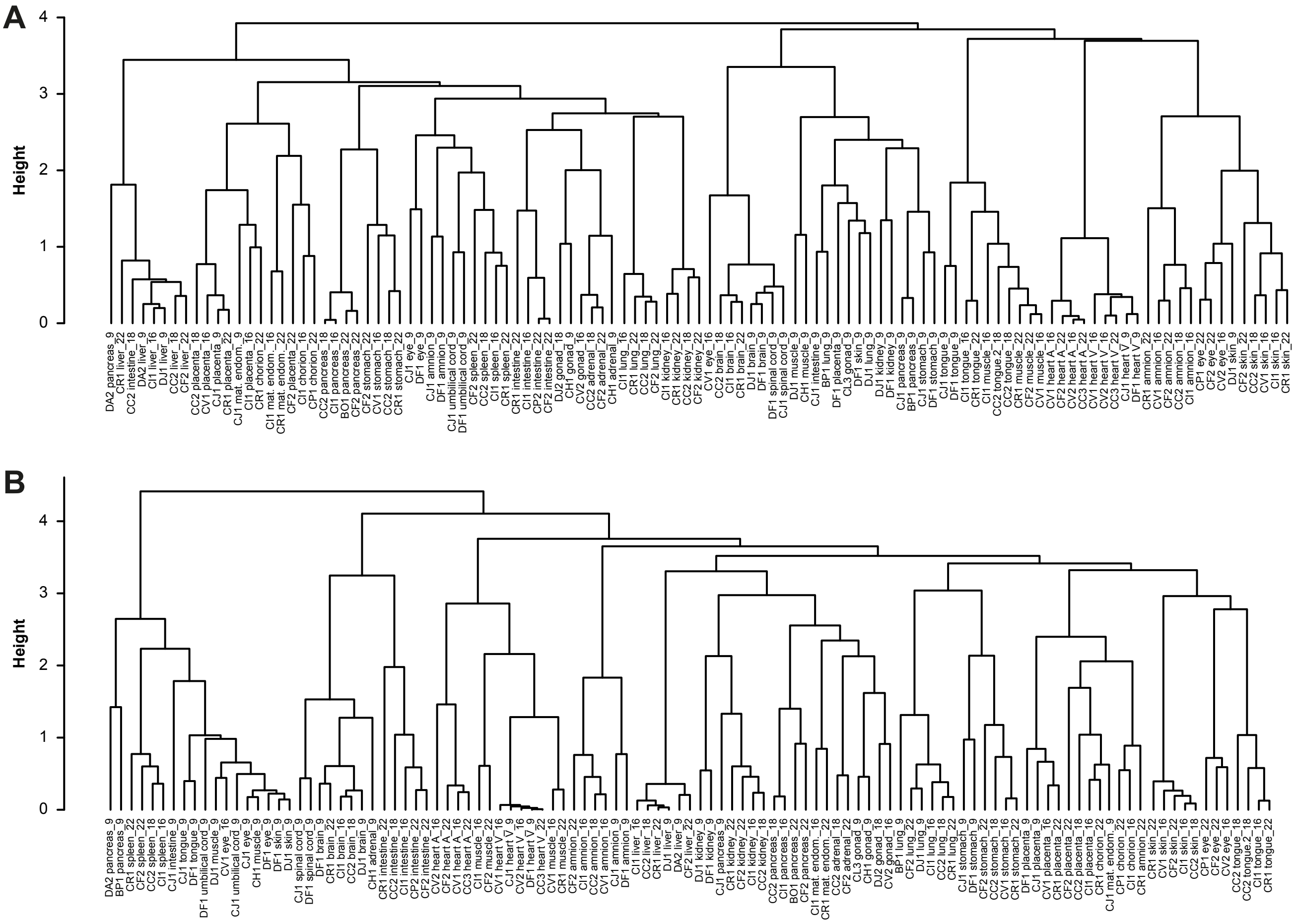

Figure S1. Hierarchical clustering of the fetal samples

(A) Dendrogram showing the hierarchical clustering of all fetal and maternal endometrium samples, based on the Pearson correlation of the expression levels of the 90 fetal classifier genes.

(B) Dendrogram showing the hierarchical clustering of all fetal and maternal endometrium samples, based on the Pearson correlation of the expression levels of the 500 most variable genes of the entire fetal data set (Top 500 fetal).

Abbreviations: mat. endom., maternal endometrium.

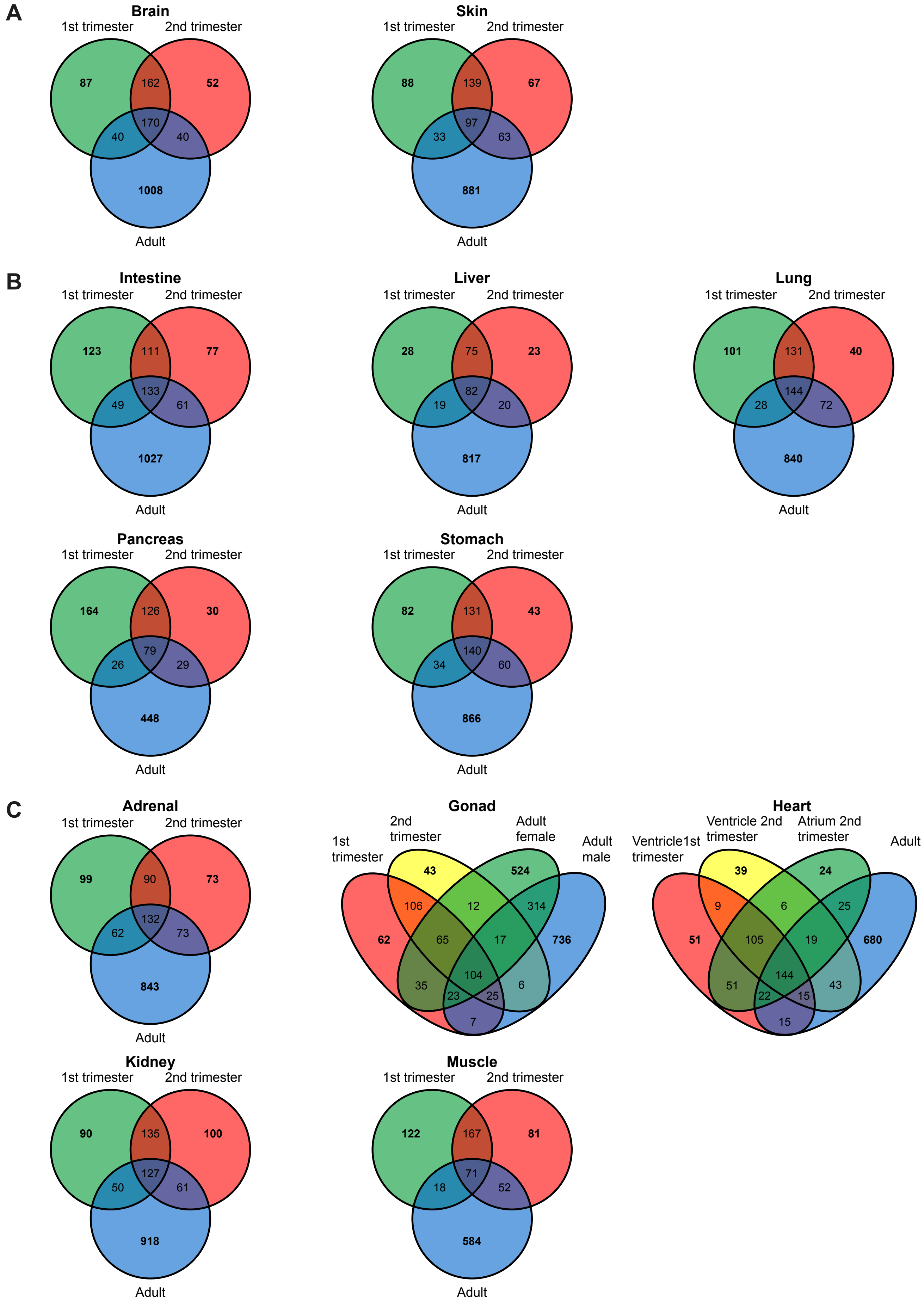

Figure S2. Stage-specific gene expression signatures per organ

Venn diagrams of commonly and uniquely highly expressed genes of the first trimester, second trimester and adult samples per organ derived mainly from (A) ectoderm, (B) endoderm, and (C) mesoderm. The genes shown here have 10x higher gene expression levels in counts per million (CPM) than the average expression of the corresponding dataset. The stage-specific genes per organ and the intersections genes are given in Table S4.

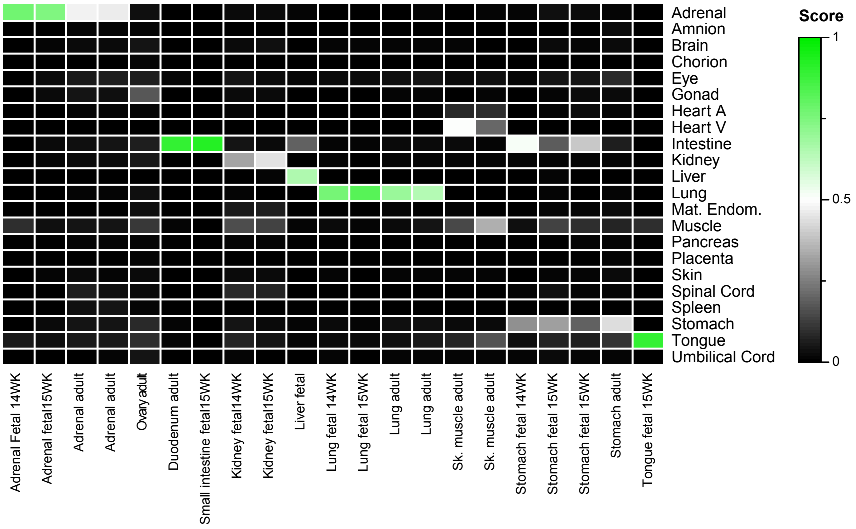

Figure S3. Prediction of the microarray human adult and fetal dataset from Nazor et al (2012)
Identity scores of 21 human adult and fetal organ/tissue samples from the microarray dataset generated by Nazor et al. (Nazor et al., 2012). The rows represent the 22 organs/tissues from the fetal training set and the columns depict the samples in the test set. The identity scores range from zero (black) to one (green). The values of all identity scores are given in Table S3.

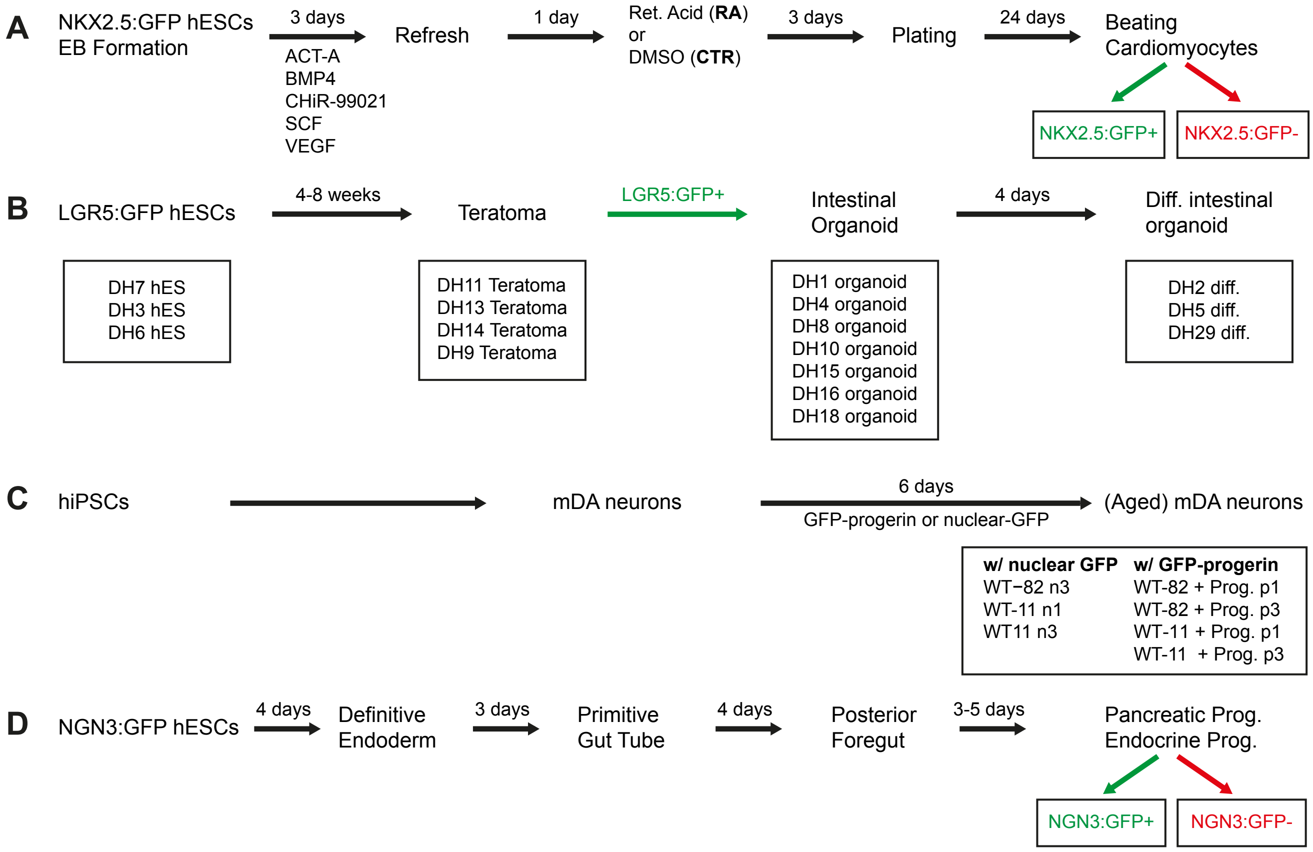

Figure S4. Schematics of the differentiation protocols used to generate hPSC-derived cardiomyocytes, intestinal organoids, dopaminergic neurons and pancreatic progenitor cells

(A) Generation of human cardiomyocytes from NKX2.5:GFP human embryonic stem cells (hESCs) (Devalla et al., 2015).

(B) Generation of intestinal organoids from LGR5:GFP human embryonic stem cells (hESCs) (Forster et al., 2014).

(C) Generation of (aged) dopaminergic neurons (mDA neurons) from human induced pluripotent stem cells (hiPSCs) (Miller et al., 2013).

(D) Generation of pancreatic progenitors from NGN3:GFP human embryonic stem cells (hESCs) (Liu et al., 2014).

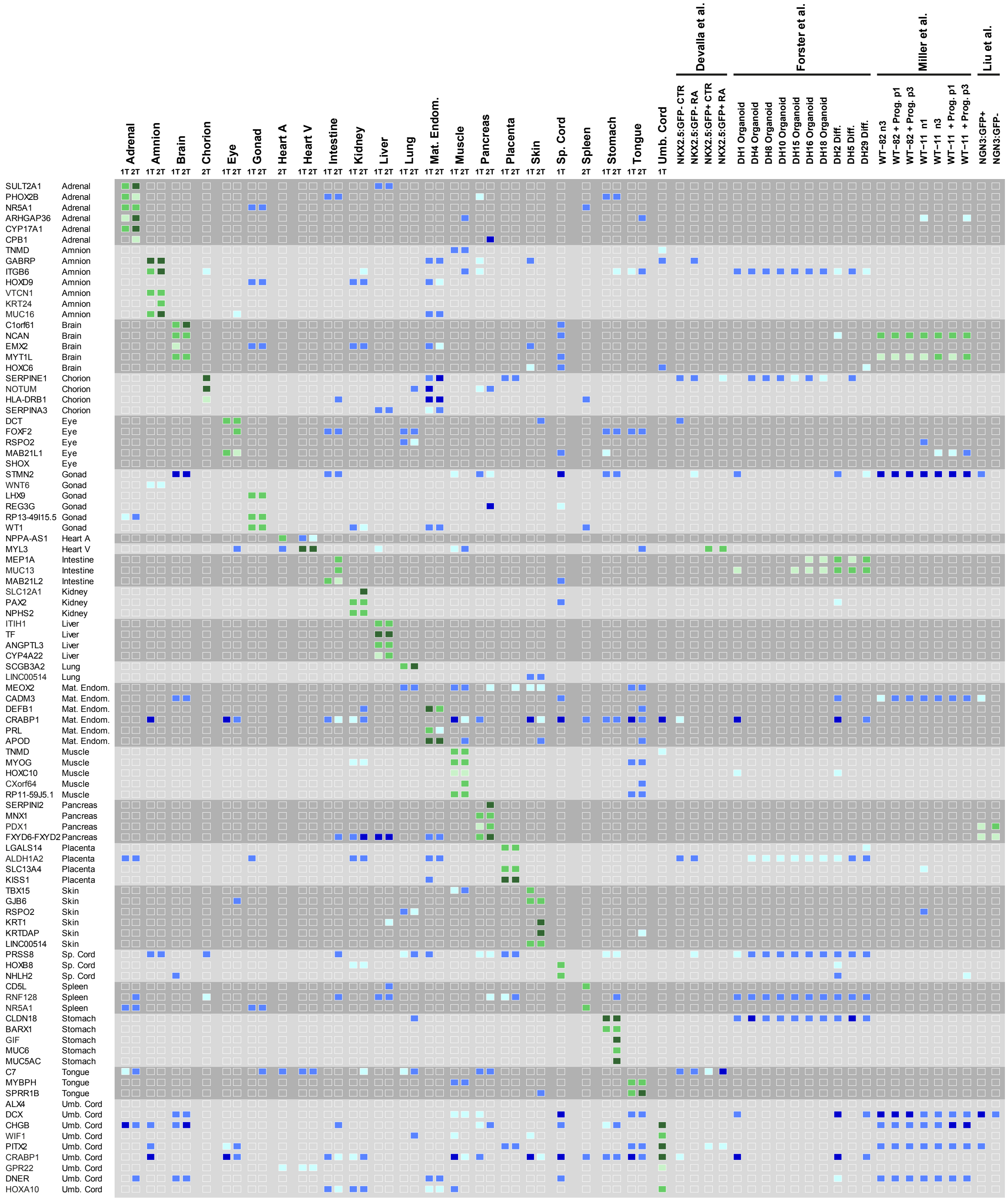

Figure S5. Expression levels of the 90 classifier genes in the hPSC-derived cardiomyocytes, intestinal organoids, dopaminergic neurons and pancreatic progenitor cells

Heatmap of the expression levels of the fetal transcriptional barcode (90 classifier genes, Figure 2) and the organ they represent during first and second trimester compared to the differentiation experiments from Figures 4 and 5 (Devalla et al., 2015; Forster et al., 2014; Liu et al., 2014; Miller et al., 2013). The expression levels (in counts per million, CPM) of the classifier genes in the corresponding tissue are depicted in green (50-100 CPM, light green; 100-1000 CPM, green; >1000 CPM, dark green), whereas the expression levels in other than the classifying tissues are displayed in blue (50-100 CPM, light blue; 100-1000 CPM, blue; >1000 CPM, dark blue).

Table S1. Characteristics of the human fetal and maternal endometrium samples

Table S2. Human fetal barcode - 90 classifier genes and the corresponding GO enrichment

Table S3. Identity scores of all test sets and the different lists of the 500 most variably expressed genes

Table S4. Specific gene expression signatures per time point per organ

SUPPLEMENTAL EXPERIMENTAL PROCEDURES

Tissue culture and immunofluorescence

Cardiomyocytes from NKX2.5:GFP hESCs were generated as described (Devalla et al., 2015) (Figure S4A). After 30 days, NKX2.5:GFP-positive cells were FACS sorted using a BD FACSAria III Cell sorter (BD Biosciences, USA) and used for library preparation. Immunofluorescence for COUP-FTII and GFP followed by imaging with confocal microscopy were described elsewhere (Devalla et al., 2015).

NGS library preparation, sequencing and data processing

The organs and tissues were homogenized using a pestle or a syringe needle followed by the QIAshredder homogenizer (Qiagen, Germany). Total RNA was isolated with the RNeasy Kit (Qiagen) including on-column DNase digestion. RNA quality was assessed with the Agilent RNA 6000 Pico or Nano Kit on an Agilent 2100 Bioanalyzer (Agilent Technologies, USA). RNA concentration was determined with the Qubit RNA BR Assay Kit on a Qubit 2.0 Fluorometer (Invitrogen, USA).

The DeepSAGE libraries (fetal organs and tissues) were generated as previously described (Mastrokolias et al., 2012). Each library (8 pM) was loaded on a v3 flowcell and sequenced on an Illumina HiSeq2000 sequencer (Illumina, USA). Sequencing data was processed with Illumina Pipeline Software version CASAVA 1.8.2, and the FASTQ files were analyzed with a set of custom Perl scripts followed by aligning the sequences against the UCSC hg19 reference genome using Bowtie short read aligner 1.0.0. Finally, the tags were annotated against Ensembl gene identifiers using BIOMART.

The strand-specific RNA-Seq libraries of the NKX2.5:GFP-positive and -negative cells were generated essentially as previously described (Parkhomchuk et al., 2009). Following amplification of the strand-specific libraries, they were paired-end sequenced (2x 75 bp) on a

single NextSeq500 Mid output lane (Illumina, USA). Base quality trimming was performed on the raw sequencing files using SICKLE 1.33. Afterwards, the sequences were aligned to a custom hg19 human genome using GSNAP release 2014-12-06. We ran GSNAP by setting the following flags: --batch (set to 4), --novelsplicing (set to 1), --npaths (set to 1), and --quiet-if-excessive. The last two flags were required so that reads map if and only if it can be mapped to a unique location in the genome. The resulting SAM file was then compressed into a BAM file, name-sorted, and indexed using SAMTOOLS 0.1.18. Finally, gene read count data were generated using HTSeq-count 0.6.1p1 using the Ensembl reference downloaded via the UCSC website (http://genome.ucsc.edu), using the non-stranded and intersection-nonempty option flags (--stranded no and --m intersection-nonempty respectively).

External Data

For the Illumina Bodymap dataset, we performed gene read counting using HTSeq-count version 0.6.1p1 using the Ensembl human genes annotation set downloaded from the UCSC website (http://genome.ucsc.edu) in December 2014. We used the non-stranded and intersection-nonempty option flags (--stranded no and --m intersection-nonempty respectively) as additional arguments to the program. As the Illumina BodyMap 2.0 data were comprised of single-end and paired-end libraries, we separated them first and performed read counting on the separated library.

SUPPLEMENTAL REFERENCES

Devalla, H.D., Schwach, V., Ford, J.W., Milnes, J.T., El-Haou, S., Jackson, C., Gkatzis, K., Elliott, D.A., Chuva de Sousa Lopes, S.M., Mummery, C.L., *et al.* (2015). Atrial-like cardiomyocytes from human pluripotent stem cells are a robust preclinical model for assessing atrial-selective pharmacology. EMBO Mol Med *7*, 394-410.

Forster, R., Chiba, K., Schaeffer, L., Regalado, S.G., Lai, C.S., Gao, Q., Kiani, S., Farin, H.F., Clevers, H., Cost, G.J., *et al.* (2014). Human intestinal tissue with adult stem cell properties derived from pluripotent stem cells. Stem cell reports *2,* 838-852.

Liu, H., Yang, H., Zhu, D., Sui, X., Li, J., Liang, Z., Xu, L., Chen, Z., Yao, A., Zhang, L., *et al.* (2014). Systematically labeling developmental stage-specific genes for the study of pancreatic beta-cell differentiation from human embryonic stem cells. Cell research *24,* 1181-1200.

Mastrokolias, A., den Dunnen, J.T., van Ommen, G.B., t Hoen, P.A., and van Roon-Mom, W.M. (2012). Increased sensitivity of next generation sequencing-based expression profiling after globin reduction in human blood RNA. BMC genomics *13*, 28.

Miller, J.D., Ganat, Y.M., Kishinevsky, S., Bowman, R.L., Liu, B., Tu, E.Y., Mandal, P.K., Vera, E., Shim, J.W., Kriks, S., *et al.* (2013). Human iPSC-based modeling of late-onset disease via progerin-induced aging. Cell stem cell *13,* 691-705.

Nazor, K.L., Altun, G., Lynch, C., Tran, H., Harness, J.V., Slavin, I., Garitaonandia, I., Muller, F.J., Wang, Y.C., Boscolo, F.S., *et al.* (2012). Recurrent variations in DNA methylation in human pluripotent stem cells and their differentiated derivatives. Cell stem cell *10,* 620-634.

Parkhomchuk, D., Borodina, T., Amstislavskiy, V., Banaru, M., Hallen, L., Krobitsch, S., Lehrach, H., and Soldatov, A. (2009). Transcriptome analysis by strand-specific sequencing of complementary DNA. Nucleic acids research *37,* e123.
